# SIRT1 mediates the excitability of spinal CaMKIIα‐positive neurons and participates in neuropathic pain by controlling Nav1.3

**DOI:** 10.1111/cns.14764

**Published:** 2024-06-03

**Authors:** Yuanzeng Wang, Yidan Zhang, Nan Ma, Wen Zhao, Xiuhua Ren, Yanyan Sun, Weidong Zang, Jing Cao

**Affiliations:** ^1^ Department of Human Anatomy, School of Basic Medical Sciences Zhengzhou University Zhengzhou Henan China; ^2^ Neuroscience Research Institute Zhengzhou University Academy of Medical Sciences Zhengzhou Henan China; ^3^ The Nursing and Health School Zhengzhou University Zhengzhou Henan China

**Keywords:** CaMKIIα^+^ neurons, Nav1.3, neuropathic pain, SIRT1

## Abstract

**Aims:**

Neuropathic pain is a common chronic pain disorder, which is largely attributed to spinal central sensitization. Calcium/calmodulin‐dependent protein kinase II alpha (CaMKIIα) activation in the spinal dorsal horn (SDH) is a major contributor to spinal sensitization. However, the exact way that CaMKIIα‐positive (CaMKIIα^+^) neurons in the SDH induce neuropathic pain is still unclear. This study aimed to explore the role of spinal CaMKIIα^+^ neurons in neuropathic pain caused by chronic constriction injury (CCI) and investigate the potential epigenetic mechanisms involved in CaMKIIα^+^ neuron activation.

**Methods:**

CCI‐induced neuropathic pain mice model, *Sirt1* ^*loxP/loxP*^ mice, and chemogenetic virus were used to investigate whether the activation of spinal CaMKIIα^+^ neurons is involved in neuropathic pain and its involved mechanism. Transcriptome sequence, western blotting, qRT‐PCR, and immunofluorescence analysis were performed to assay the expression of related molecules and activation of neurons. Co‐immunoprecipitation was used to observe the binding relationship of protein. Chromatin immunoprecipitation (ChIP)‐PCR was applied to analyze the acetylation of histone H3 in the *Scn3a* promoter region.

**Results:**

The expression of sodium channel Nav1.3 was increased and the expression of SIRT1 was decreased in the spinal CaMKIIα^+^ neurons of CCI mice. CaMKIIα neurons became overactive after CCI, and inhibiting their activation relieved CCI‐induced pain. Overexpression of SIRT1 reversed the increase of Nav1.3 and alleviated pain, while knockdown of SIRT1 or overexpression of Nav1.3 promoted CaMKIIα^+^ neuron activation and induced pain. By knocking down spinal SIRT1, the acetylation of histone H3 in the *Scn3a* (encoding Nav1.3) promoter region was increased, leading to an increased expression of Nav1.3.

**Conclusion:**

The findings suggest that an aberrant reduction of spinal SIRT1 after nerve injury epigenetically increases Nav1.3, subsequently activating CaMKIIα^+^ neurons and causing neuropathic pain.

## Graphical abstract

The downregulation of SIRT1 in the SDH, following CCI, leads to increased histone H3 acetylation in the *Scn3a* promoter region. This promotes the expression of Nav1.3, which subsequently causes CaMKIIα^+^ neurons activation, resulting in pain. Alleviating pain by reversing SIRT1 downregulation can be a potential therapeutic target for local spinal cord analgesics.

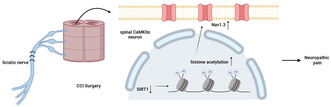

## 1 INTRODUCTION

Neuropathic pain originates from peripheral or central nerve lesions, is caused by multiple factors, and has an estimated prevalence of 7%–10% in the general population.^1^ The treatment options for neuropathic pain were diverse, including drug therapy, physical therapy, psychological therapy, and neuromodulation therapy. Drug therapy was the first choice, mainly including antidepressants, antiepileptics, opioids, etc.^2^ Despite the variety of treatments available, the effectiveness of managing neuropathic pain remains suboptimal. Scores of patients suffer from significant side effects and unstable efficacy. For example, prolonged use of nonsteroidal anti‐inflammatory drugs (NSAIDs) can lead to gastric ulcers, and morphine use may lead to bowel dysfunction and tolerance.^3^ One of the main reasons for the restricted clinical treatment of neuropathic pain is the uncertainty of its pathogenesis. We need to have a more complete understanding of the mechanism behind the occurrence and development of neuropathic pain to find safer and more effective treatment targets.

The spinal cord serves as the central hub for integrating and transmitting pain signals, with sensitized spinal neurons considered a key factor in neuropathic pain development.^4^ Interneurons in layers I, II, and III of the SDH are categorized into excitatory and inhibitory neurons, with excessive stimulation of excitatory interneurons and subsequent signal transmission to projection neurons believed to be crucial in pain generation.^5^ CaMKII is a multisubunit enzyme composed of four different genes: CaMKIIα–CaMKIIδ. Among these, CaMKIIα is particularly abundant in the central nervous system and is the most prevalent protein at excitatory synapses.^6^, ^7^ Activation of NMDA receptors in neurons leads to CaMKII activation, which in turn induces long‐term potentiation, a crucial aspect of central sensitization.^8^ Studies have shown that CaMKIIα expression and activity are increased in the superficial layer of the SDH and dorsal root ganglia in chronic pain. Inhibitors of CaMKIIα have been observed to reduce pain‐related behaviors in a dose‐dependent manner.^9^, ^10^ However, further researches are required to elucidate the mechanisms of action of spinal CaMKIIα^+^ neurons in chronic pain.

The activation of ion channels is known to play a crucial role in maintaining neuronal excitability, particularly in the context of neuropathic pain.^11^ The heightened expression, density, and functionality of excitatory ion channels, such as voltage‐gated sodium channels, have been linked to increased neurotransmitter release, excitability, and ectopic firing of sensory neurons.^12^ Following nerve injury, there is a rapid and prolonged increase in Nav1.3 expression in the dorsal root ganglion (DRG), leading to ectopic firing and the development of abnormal mechanical pain.^13^, ^14^ Studies have shown that knockdown of Nav1.3 in DRG can mitigate mechanical allodynia^15^ and diabetic neuropathic pain in rats.^16^ Additionally, peripheral nerve injury can also result in the upregulation of Nav1.3 in spinal neurons, and decreasing its expression has been found to alleviate pain‐related behaviors.^17^ However, the specific subtypes of spinal neurons that increasingly express Nav1.3 and the underlying mechanisms contributing to neuropathic pain remain unreported.

Emerging evidence indicates that epigenetic alterations contribute to the development of neuropathic pain.^18^ SIRT1, a member of the sirtuins family, acts as a histone deacetylase, exerting a transcriptional silencing effect.^19^ Studies have shown reduced levels of SIRT1 in various pain models, indicating that its activation may be pivotal in alleviating chronic pain.^20^ Conversely, SIRT1 deficiency has been linked to cardiac disorders resulting from impaired regulation of sodium and calcium ions.^21^ In this study, we aimed to investigate the involvement of spinal CaMKIIα‐positive (CaMKIIα^+^) neurons in neuropathic pain caused by chronic constriction injury (CCI) surgery and its potential epigenetic mechanisms.

## 2 MATERIALS AND METHODS

### 2.1 Animals

Adult male C57BL/6, Ai14 (Rosa26‐LSL Tdtomato), GAD‐GFP mice, CaMKIIα‐Cre, and *Sirt1* ^*loxP/loxP*^ male mice aged 8–10 weeks were used in this study. C57BL/6 mice were purchased from the Experimental Animal Center of Zhengzhou University. *Sirt1* ^*loxP/loxP*^ mice were obtained from Jianyuan Zhao's group affiliated with Shanghai Jiao Tong University School of Medicine. GAD‐GFP mice, CaMKII‐Cre, and Ai‐14 mice were purchased from the Shanghai Model Organisms Center. In order to identify CaMKIIα^+^ neurons, CaMKII‐Cre male mice were crossed with Ai14 tdTomato female mice to obtain CaMKII‐Cre, and Ai14 mice with tdTomato fluorescence in Cre‐expressing cells (CaMKII‐Cre; tdTomato). All animals were kept under controlled conditions (25 ± 1°C, 12‐h light–dark cycles) with access to food and tap water ad libitum. The experimental procedures used in this study were approved by the Animal Center of Henan Province. The approval was granted in accordance with the guidelines issued by the Animal Care Committees of Zhengzhou University and the Life Sciences Ethics Review Committee of Zhengzhou University.

### 2.2 Chronic constriction injury (CCI) surgery

The CCI surgery was carried out as described previously with minor modifications.^22^ Mice were anesthetized with inhaled isoflurane in 100% oxygen (induced at 4%–5%; maintained at 2%–3%). In the CCI group, the left sciatic nerve was exposed and three 6–0 chromic catgut ligatures were loosely tied around the nerve with 1.5 mm spacing. The sham group underwent an identical procedure but without the ligatures. The animals were randomly assigned to either the CCI or sham group.

### 2.3 Behavioral assessment

Von Frey filaments (Stoelting, Kiel, Wl, USA) were utilized to assess the paw withdrawal threshold (PWT) to mechanical stimuli. Prior to testing, the animals were acclimated in a plastic box on a metal mesh for 30 min. The filaments, ranging in bending forces from 0.04 to 2 g, were applied to the mid‐plantar surface of the hind paw with sufficient force to bend the filament for 3 s. Positive responses, such as paw lifting, claw licking, or signs of escaping, were recorded. If no response was observed, a filament with a higher force was used. Conversely, upon a positive response, a filament with a lower force was applied. The experimenter conducting the tests was blinded to all treatments. The paw withdrawal threshold was determined using the “up‐down” method.^23^

The paw withdrawal latencies (PWL) to heat stimuli in mice were measured using heat‐flux radiometer (UGO BASILE S.R.L., Italy). The mice were acclimated on a glass plate inside a plastic box for 30 min before the test. Subsequently, a radiant heat beam was applied to the left hind paw until a positive reaction was elicited. The latency (maximum of 20 s) of flinching, licking, or jumping behavior was then documented. Each measurement was conducted thrice, and the mean value was subsequently calculated.

### 2.4 Intraspinal cord microinjection

The SIRT1 lentiviral activation particles (LV‐SIRT1; sc‐425704‐LAC), Nav1.3 lentiviral activation particles (LV‐Nav1.3; sc‐422820‐LAC), control lentiviral activation particles (LV‐NC; sc‐437282), Nav1.3 shRNA lentiviral particles (LV‐Nav1.3 shRNA; sc‐42647‐V), and control shRNA lentiviral particles (LV‐control shRNA; sc‐108080) were purchased from Santa Cruz Biotechnology Co., Ltd. (Shanghai, China). Adeno‐associated virus (AAV) containing a gene‐encoding hM4Di under the control of the CaMKIIα promoter (pAAV‐CaMKIIα‐hM4D(Gi)‐EGFP or pAAV‐CaMKIIα‐hM4D(Gi)‐mCherry) were purchased from HanBio (Shanghai, China). The viral vector microinjection procedure followed the methods described in previous studies.^24^, ^25^ Mice were anesthetized with isoflurane inhalation in 100% oxygen and positioned prone on the operating table. Following a midline incision at the base of the rib cage, the spinal cord was exposed by separating the overlying fascia and removing the dorsal part of the vertebra using laminectomy forceps. Next, a glass capillary connected to a glass microinjector (2 μL; RWD Life Science) was inserted 500 μm to the left side of the spinal cord midline at a depth of 250 μm, and lentiviral activation particles were injected in a 250 nL suspension at a rate of 50 nL/min. The capillary was left in place for 10 min post‐injection.

### 2.5 Western blotting analysis

The mice were deeply anesthetized with 5% isoflurane and promptly decapitated. The dorsal horn of the spinal segment was isolated using a lysis buffer comprising 10 mM Tris (pH 7.5), 1 mM PMSF, 5 mM MgCl_2_, 5 mM EGTA, 1 mM EDTA, 1 mM DTT, 40 mM leupeptin, and 250 mM sucrose with added protease and phosphatase inhibitors. The protein concentration was determined using the BCA Protein Assay Kit (Solarbio, Beijing, China). Equal amounts of protein samples were then separated using 8% sodium dodecyl sulfate (SDS)‐polyacrylamide gel electrophoresis (CoWin Biosciences, Beijing, China). The protein was then transferred onto a PVDF membrane (Millipore Corp., Billerica, MA, USA) and blocked with 5% bovine serum albumin (Solarbio) at room temperature. The membrane was incubated with appropriate primary antibodies including anti‐SIRT1 (1:1000, ab110304, Abcam, UK), anti‐Nav1.3 (1:200, #ASC‐004, Allomone labs, Israel), anti‐GAPDH (1:5000, ab8245, Abcam, UK), antihistone H3 (1:1000, #9715, Cell Signaling Technology, USA), antiacetylated lysine (1:1000, #9441, Cell Signaling Technology, USA), and antiacetyl‐histone H3 (1:1000, #3782221, EMD Millipore Corp, USA). FluorChem Protein Simple (AlphaImager ProteinSimple, San Jose, CA, USA) was applied to detect immunoreactive bands. The bands were then quantified with computer‐assisted imaging analysis system (ImageJ).

### 2.6 Quantitative real‐time reverse transcription PCR (qRT‐PCR)

Dorsal horn tissue total RNA was isolated using the Axyprep total RNA isolation kit. RNA concentration and purity were assessed with a spectrophotometer (Thermo Fisher Scientific, USA) at 260 and 280 nm. Subsequently, cDNA was synthesized from mRNA using the RevertAid First Strand cDNA Synthesis Kit (Thermo Fisher Scientific, USA). Quantitative PCR was conducted using the iQ5 real‐time PCR detection system (Bio‐Rad) and the SYBR Green qPCR Master Mix kit (Thermo Fisher Scientific, USA). Each sample was analyzed in triplicate. The 2^−△△CT^ method was employed to quantify the relative expression ratio of mRNA.^26^ The specific primer sequences used were as follows: GAPDH (forward, 5′‐TCGGTGTGAACG GATTTGGC‐3′ and reverse, 5′‐TCCCATTCTCGGCCTTGACT‐3′); Sirt1 (forward, 5′‐CCAGACCTCCCAGACCCTCAAG‐3′ and reverse, 5′‐GTGACACAGAGA CGGCTGGAAC‐3′); and *Scn3a* (forward, 5′‐ACTGTGTTCTGTCTGAGCGTCTTTG‐3′ and reverse, 5′‐ACTGTGTTCTGTCTGAGCGTCTTTG‐3′).

### 2.7 Immunohistochemistry

The mice were deeply anesthetized with 5% isoflurane, followed by cardiac perfusion using 0.9% physiological saline and 4% paraformaldehyde in PBS. The spinal cord slices underwent three 10‐min washes with PBS after dewaxing, followed by a 2‐h incubation at room temperature in a blocking buffer containing 10% goat serum and 0.3% Triton X‐100. The sections were subsequently incubated with primary antibodies overnight at 4°C, including anti‐SIRT1 (1:100, #8469, Cell Signaling Technology, USA), anti‐Nav1.3 (1:100, #ASC‐004, Allomone labs, Israel), anti‐GFAP (1:200, #3670, Cell Signaling Technology, USA), anti‐GFAP (1:200, #80788, Cell Signaling Technology, USA), anti‐Iba1 (1:200, ab283319, Abcam, UK), anti‐Iba1 (1:100, ab178847, Abcam, UK), anti‐NeuN (1:200, ab177487, Abcam, UK), anti‐NeuN (1:500, #94403, Cell Signaling Technology, USA), anti‐CaMKIIα (1:100, #50049, Cell Signaling Technology, USA), anti‐CaMKIIα (1:100, ab131468, Abcam, UK), and anti‐VgluT2 (1:100, Cat#AGC‐036, Alomone, Israel). Subsequently, the sections were rinsed three times (10 min each time) with PBS and incubated with a fluorescent dye‐conjugated secondary antibody for 2 h at room temperature. After washing with PBS for three times (10 min each), the sections were incubated with DAPI. Further analyses were conducted using ImageJ software by an observer blind to the conditions.

### 2.8 Chromatin immunoprecipitation (ChIP)

The ChromaFlash™ High‐Sensitivity ChIP Kit Epigentek (Catalog# P‐2027) was utilized to conduct a chromatin immunoprecipitation (ChIP) assay on spinal tissues, in accordance with the manufacturer's instructions. Mice spinal cord tissue was homogenized in an enzyme‐free tube with PBS buffer and protease inhibitor. The homogenates were cross‐linked with 37% formaldehyde for 10 min at room temperature, followed by de‐crosslinking using 1.25 M glycine solution for 5 min on ice. After low‐temperature centrifugation, the DNA–protein complexes were obtained by sonication, washing with PBS buffer, and addition of WLB solution. The DNA–protein complexes were then incubated with the antiacetyl H3 antibody in the wells overnight at 4°C. Following the incubation, the wells were washed with WB solution, and RNase A solution was added, followed by a 30‐min reaction at 42°C. Protein kinase K was subsequently added, and the reaction continued for 45 min at 60°C. The purified DNA in the wells was transferred to enzyme‐free EP tubes and heated in a metal heating pot at 95°C for 15 min. PCR amplification was performed, followed by electrophoresis on an agarose gel, and chemiluminescence imaging was conducted after electrophoresis. Immunoprecipitated DNA fractions were then subjected to quantitative real‐time PCR using primers specific to the mouse *Scn3a*. We used the following primers: *Scn3a*, 5′‐AGGTATTCAGATCAGAGGTTGTC‐3′ and 5′‐GCCTTCCGAAGAGATGGAGAATAA‐3′ (−529 to −139 bp).

### 2.9 Co‐immunoprecipitation

Co‐immunoprecipitation was performed using the Immunoprecipitation Kit (PK10007, Proteintech, USA). The spinal cord tissue was thoroughly homogenized at low temperature with IP Lysis buffer. The homogenate was sonicated and left to stand for 60 min, with inversion every 10 min. Following protein concentration measurement, protein A Sepharose beads were added to 1.5 mg of total protein along with specific primary antibodies. Negative controls were treated with IgG of the same species. The samples were rotated and incubated overnight at 4°C. Upon completion of the incubation, the immune complexes were eluted and subsequently heated in a boiling water bath for 5 min after the addition of sample buffer. The eluted complexes were then subjected to analysis by western blotting.

### 2.10 Statistical analysis

Data in this study were presented as mean ± SEM and analyzed using GraphPad Prism 8.0. (GraphPad Inc., San Diego, CA, USA). Normality was initially assessed for all data before analysis. Shapiro–Wilk test was applied to test the normal distribution of the data. If the data are normally distributed with uniform variance, Student's *t*‐test was performed when only two groups were applied, one‐way analysis of variance (ANOVA) when three or more groups were applied, and two‐way repeated measures ANOVA to compare two variables in two or more groups. Tukey's multiple comparisons test was performed after ANOVA. Otherwise, Mann–Whitney *U* and Kruskal–Wilcoxon tests were performed if the data were not normally distributed. *p* < 0.05 was considered statistically significant.

## 3 RESULTS

### 3.1 Inhibition of CCI‐induced activation of CaMKIIα ^+^ neurons alleviates pain

**FIGURE 1:**
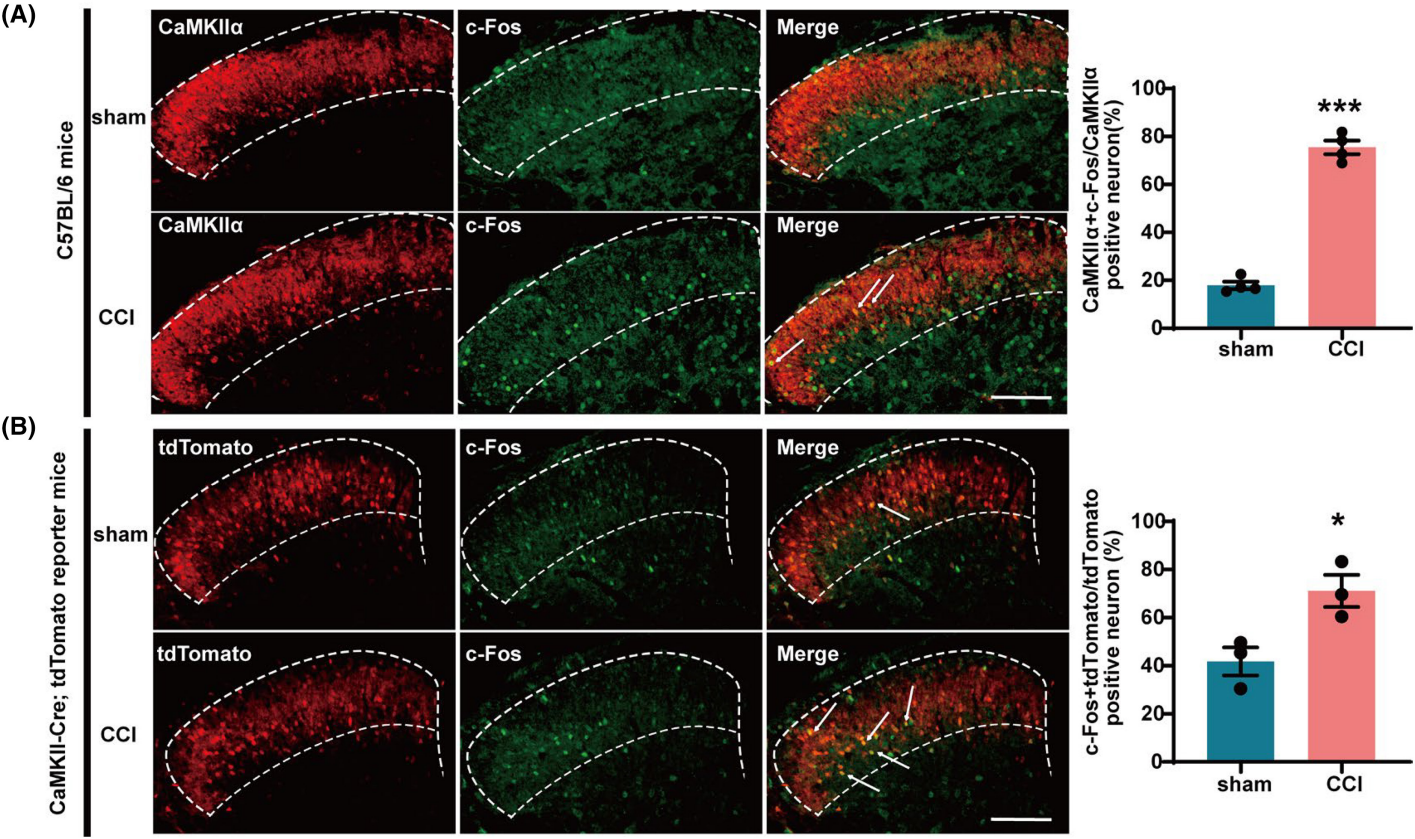
CCI surgery induced CaMKIIα^+^ neuron activation. (A) The percentage of CaMKIIα protein co‐labeled with c‐Fos increased in the SDH of CCI mice. ***p < 0.001 compared with sham. Scale bars = 200 μm. (B) The percentage of tdTomato‐labeled CaMKIIα^+^ neurons co‐labeled with c‐Fos increased in the SDH of CCI mice. *p < 0.05 compared with sham. Scale bars = 200 μm.

We established a chronic constriction injury model of the sciatic nerve to simulate the clinical symptoms of nerve compression caused by trauma. Immunofluorescence showed that the proportion of CaMKIIα protein co‐labeled with c‐Fos in the SDH of C57BL/6 mice on day 14 after CCI increased significantly compared with sham group (Figure 1A). To test whether CCI surgery induced CaMKIIα^+^ neuron activation, CaMKII‐Cre; tdTomato reporter mice were used to perform immunofluorescence in tdTomato‐labeled CaMKIIα^+^ neurons in the spinal. As expected, we found that the positive expression of c‐Fos in CaMKIIα^+^ neurons increased remarkably in the CCI group compared to the sham group on the 14th postoperative day (Figure 1B). These results indicate that nerve injury induced the activation of CaMKIIα^+^ neurons.

**FIGURE 2:**
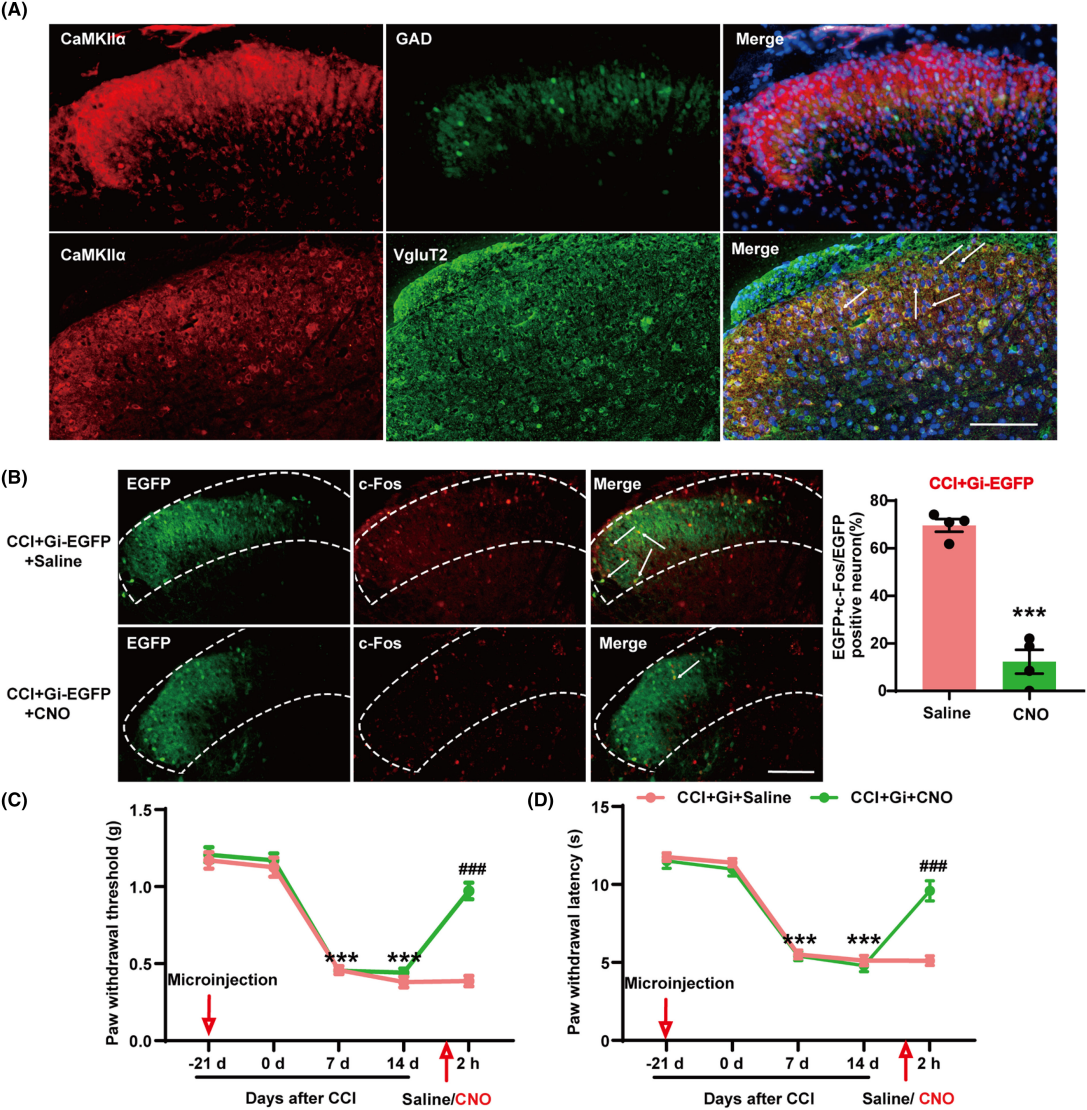
Inhibition of SDH CaMKIIα^+^ neurons alleviated CCI‐induced pain. (A) Co‐detection of CaMKIIα and VgluT2 and/or GAD in the SDH of mice. (B) The percentage of CaMKIIα^+^ neurons co‐labeled with c‐Fos in the SDH of CCI mice was reduced after activation of pAAV‐CaMKIIα‐hM4D(Gi)‐EGFP by CNO. ***p < 0.001 compared with CCI + Gi‐EGFP + Saline. (C) The paw withdrawal threshold was significantly increased after inhibition of CaMKIIα^+^ neurons in CCI mice. (D) The paw withdrawal latency was significantly increased after inhibition of CaMKIIα^+^ neuron activation in CCI mice. ***p < 0.001 compared with CCI + Gi‐EGFP + Saline 0 day ^*###*^ *p* < 0.001 vs. CCI + Gi‐EGFP + Saline. SDH, spinal dorsal horn.

Next, we investigated the role of spinal CaMKIIα^+^ neurons in pain processing. To determine whether SDH CaMKIIα is expressed in excitatory or inhibitory neurons, we used double‐labeling experiments to study its localization relative to vesicular glutamate transporter‐2 (VgluT2) and/or glutamic acid decarboxylase (GAD). VgluT2 is the most common marker of excitatory glutamatergic neurons, and GAD is a GABAergic neuron marker. Using GAD‐GFP mice and immunostaining for Vglut2, we found that CaMKIIα immunoreactivity was primarily co‐localized with Vglut2, and lacking co‐localization with GAD neurons (Figure 2A). We then used a chemogenetic virus to further investigate whether inhibition of CaMKIIα^+^ neuron activation could alleviate pain. We microinjected pAAV‐CaMKIIα‐hM4D(Gi)‐EGFP unilaterally into the SDH of mice 21 days before CCI surgery. The site of the virus injection and the surgical side for CCI are on the same side. On the 14th day following CCI surgery, behavioral tests were performed 2 h before and after CNO treatment. We observed that the co‐labeling ratio of c‐Fos and EGFP in the SDH of CCI mice injected with CNO was significantly decreased (Figure 2B). This indicated an inhibition of CCI‐induced activation of CaMKIIα^+^ neurons. At the same time, 14 days after CCI, the mechanical and thermal pain thresholds of CCI mice that had undergone the activation of hM4D with CNO were significantly higher than those of CCI mice injected with saline (Figure 2C,D), indicating that CCI‐induced pain was relieved. These results suggest that inhibition of spinal CaMKIIα^+^ neurons can alleviate pain.

### 3.2 The expression of Nav1.3 is increased in the SDH of CCI mice

**FIGURE 3:**
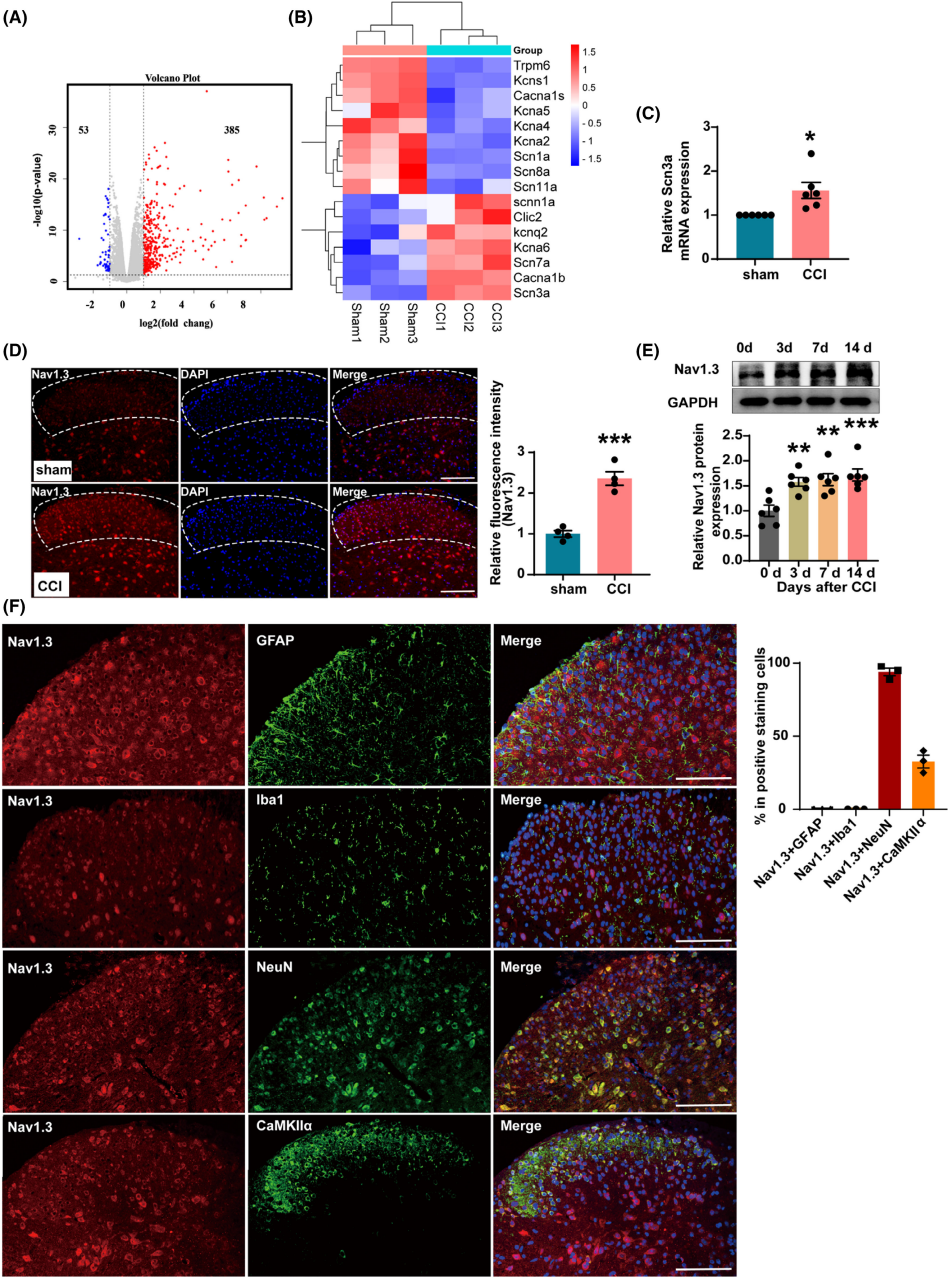
Nav1.3 was upregulated in the SDH of CCI mice. (A) Volcano plot showing the overall distribution of upregulated and downregulated mRNAs between the sham group and the CCI group mice. (B) Heatmap showing the differential expression of ion channel genes in the SDH of the sham and CCI mice. (C) The mRNA level of *Scn3a* was increased in the SDH of CCI mice. *p < 0.05 compared with sham. (D) Mean fluorescence intensity of the Nav1.3 immunostaining cells increased significantly in the ipsilateral SDH of CCI mice. ***p < 0.001 compared with sham. (E) Representative western blot and corresponding graphs showing that Nav1.3 protein level was increased in the SDH of CCI mice. **p < 0.01 and ***p < 0.001 compared with 0 day. (F) The photographs showed the double staining between NeuN or GFAP or Iba1 or CaMKIIα with Nav1.3 in SDH of mice. SDH: spinal dorsal horn. Scale bars = 200 μm.

To explore the key regulatory molecules involved in the development of nerve injury‐induced pain and their regulatory mechanisms, we performed transcriptome sequencing of the lumbar SDH tissue on the operated side of mice in the sham 14‐day group and CCI 14‐day group (Figure 3A). As neuronal excitability is closely linked to various ion channels, we analyzed the differentially expressed genes related to ion channels. Our findings showed a significant increase in the transcript level of the *Scn3a* gene, which encodes the voltage‐gated sodium channel Nav1.3 after CCI (Figure 3B). We also performed q‐PCR experiments and confirmed that the mRNA level of *Scn3a* was significantly elevated in the SDH of CCI mice (Figure 3C). Additionally, immunofluorescence staining results showed that the fluorescence signal intensity of Nav1.3 in the SDH of mice was significantly increased after CCI (Figure 3D). Likewise, western blot showed that the Nav1.3 protein significantly increased in SDH on days 3, 7, and 14 after CCI (Figure 3E). We then examined the localization of Nav1.3. The results showed that Nav1.3 co‐localized with NeuN (Neuron) and Nav1.3 hardly showed any overlap staining with either the astrocytic marker GFAP (glial fibrillary acidic protein) or the microglial marker Iba1 (ionized calcium‐binding adapter molecule 1). Further observation revealed that Nav1.3 was co‐labeled with CaMKIIα^+^ neurons (Figure 3F). Taken together, these results suggest that the increased expression of Nav1.3 in CaMKIIα^+^ neurons may be a critical factor in pain induction.

### 3.3 Downregulation of SDH Nav1.3 in CCI mice alleviated pain, whereas overexpression of Nav1.3 induced pain

**FIGURE 4:**
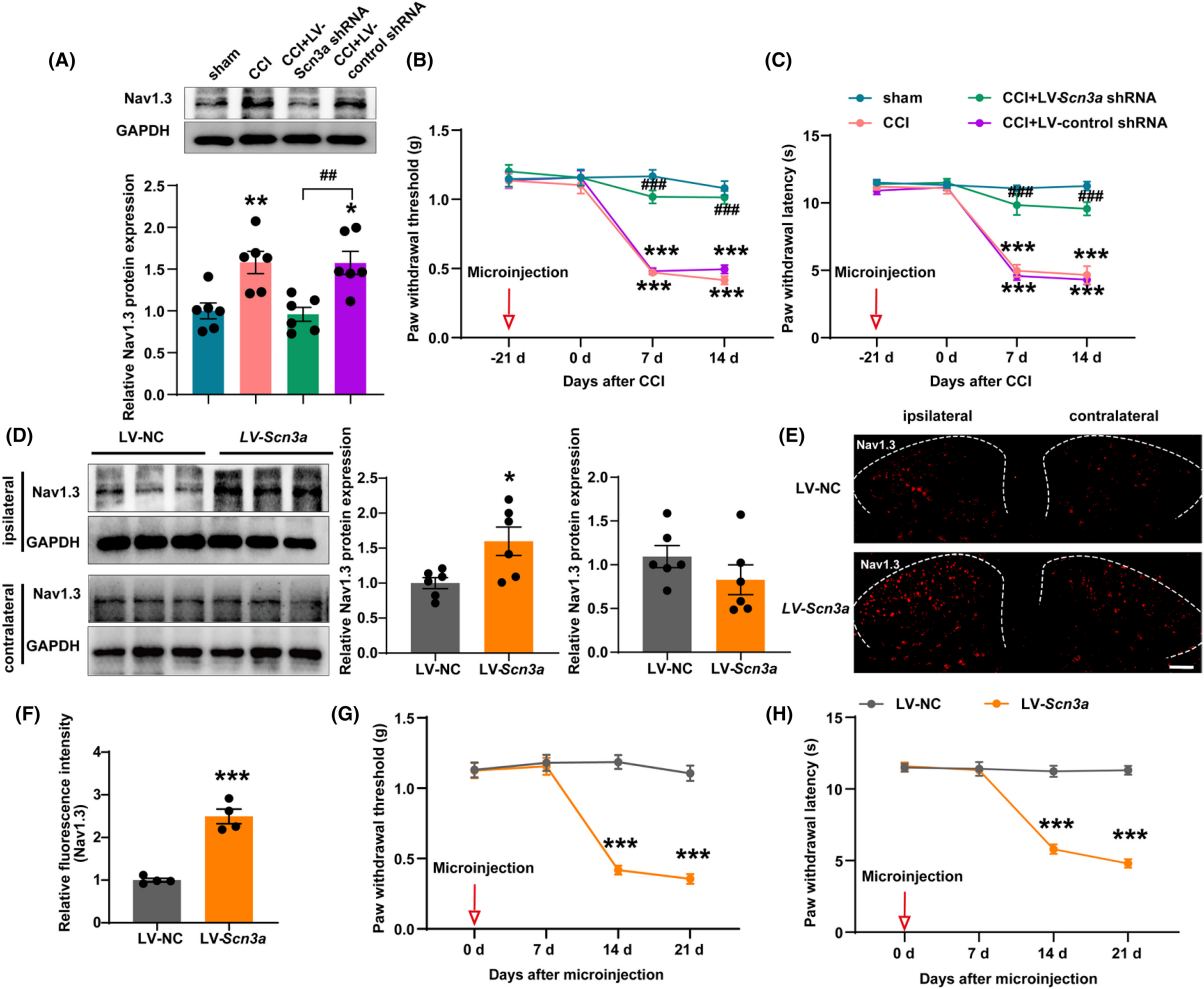
Knockdown of Nav1.3 in SDH reversed mechanical and thermal hyperalgesia of CCI mice while overexpression of Nav1.3 decreased nociceptive thresholds. (A) Representative western blot and corresponding graphs showing that Nav1.3 protein level decreased in the SDH of CCI mice after microinjection of LV‐*Scn3a* shRNA. **p < 0.01 compared with sham; ^*##*^ *p* < 0.01 compared with CCI + LV‐NC. (B, C) Microinjection of LV‐*Scn3a* shRNA into the ipsilateral SDH increased paw withdrawal threshold and paw withdrawal latency of CCI mice. ***p < 0.001 compared with sham; ^*###*^ *p* < 0.001 compared with CCI + LV‐NC. (D) Representative western blot and corresponding graphs show that Nav1.3 protein level increased in the ipsilateral SDH of naive mice after microinjection of LV‐*Scn3a*. *p < 0.05 compared with LV‐NC. (E, F) Mean fluorescence intensity of the Nav1.3 immunostaining cells increased significantly in the ipsilateral SDH of naive mice after microinjection of LV‐*Scn3a*. ***p < 0.001 compared with LV‐NC. Scale bars = 200 μm. (G, H) Microinjection of LV‐*Scn3a* into the SDH decreased paw withdrawal threshold and paw withdrawal latency of naive mice. ***p < 0.001 compared with LV‐NC. SDH: spinal dorsal horn.

To further validate the role of Nav1.3 in the pain process, we injected LV‐*Scn3a* shRNA lentivirus into the ipsilateral SDH of CCI mice to inhibit the expression of Nav1.3 and injected lentivirus LV‐*Scn3a* into the SDH of naive mice to overexpress Nav1.3. Firstly, we microinjected LV‐*Scn3a* shRNA and LV‐control shRNA into the ipsilateral SDH of mice 21 days before CCI. The site of the virus injection and the surgical side for CCI are on the same side. After 21 days of recovery, the mice then underwent CCI surgery. The western blot analysis showed a significant decrease in the Nav1.3 protein expression in the CCI group that received LV‐*Scn3a* shRNA injection (Figure 4A). Compared to CCI mice injected with LV‐control shRNA, the paw withdrawal threshold and paw withdrawal latency of CCI mice with Nav1.3 downregulation were significantly increased (Figure 4B,C).

Subsequently, we injected lentivirus LV‐*Scn3a* into unilateral SDH of naive mice to overexpress Nav1.3. The western blot analysis showed a significant increase in the Nav1.3 protein expression in the ipsilateral SDH with injection of LV‐*Scn3a*. However, Nav1.3 expression levels in the contralateral SDH were not significantly different between the LV‐NC and the LV‐*Scn3a* groups (Figure 4D). Additionally, the immunofluorescence staining depicted significantly increased fluorescence signal intensity of Nav1.3 in the SDH of LV‐*Scn3a*‐injected mice group, indicating successful transfection of the lentivirus into the ipsilateral SDH (Figure 4E,F). Compared with the mice in the LV‐NC group, the paw withdrawal threshold and paw withdrawal latency of mice with Nav1.3 overexpression decreased significantly at days 14 and 21 (Figure 4G,H). Overall, these results suggest that Nav1.3 in the SDH plays a crucial role in neuropathic pain. Inhibition of its expression relieves pain, while overexpression induces pain.

### 3.4 Chemogenetic inhibition of CaMKIIα ^+^ neurons attenuated pain induced by Nav1.3 overexpression in the SDH

**FIGURE 5:**
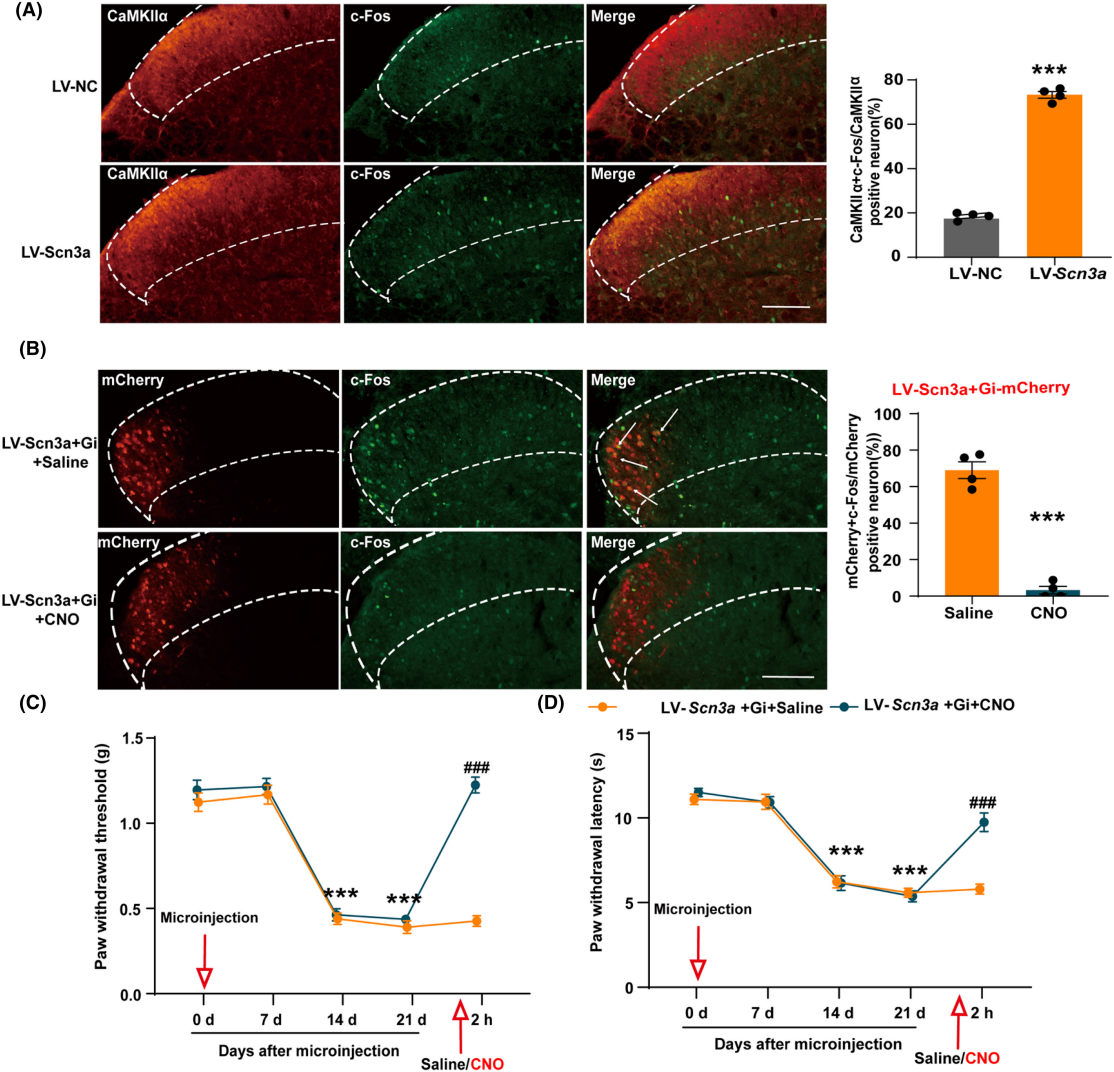
Inhibition of CaMKIIα^+^ neuron activation reversed the hyperalgesia induced by Nav1.3 overexpression in the SDH. (A) The percentage of CaMKIIα^+^ neurons co‐labeled with c‐Fos increased in the SDH of mice after microinjection of LV‐*Scn3a*. ***p < 0.001 compared with LV‐NC. Scale bars = 200 μm. (B) Activation of chemogenetic viruses by CNO reduced the percentage of CaMKIIα^+^ neurons co‐labeled with c‐Fos in the SHD of mice microinjected with LV‐*Scn3a*. ***p < 0.001 compared with LV‐*Scn3a* + Gi + Saline. Scale bars = 200 μm. (C, D) Activation of chemogenetic viruses by CNO increased paw withdrawal threshold and paw withdrawal latency of mice microinjected with LV‐*Scn3a*. ***p < 0.001 compared with LV‐*Scn3a* + Gi + CNO 21 d; ^*###*^ *p* < 0.001 compared with LV‐*Scn3a* + Gi + Saline. SDH: spinal dorsal horn.

To further investigate the relationship between Nav1.3 and CaMKIIα^+^ neurons, we examined the activity of CaMKIIα^+^ neurons in the SDH after injection of lentivirus LV‐*Scn3a*. We found that the proportion of c‐Fos expression in CaMKIIα neurons was enhanced in SDH of LV‐*Scn3a*‐injected mice than control group, indicating that overexpression of Nav1.3 increased the excitability of CaMKIIα neurons (Figure 5A). Subsequently, we further verified whether pain was induced through CaMKIIα^+^ neuron activation after Nav1.3 overexpression by injecting LV‐*Scn3a* along with the chemogenetic virus pAAV‐CaMKIIα‐hM4D(Gi)‐mCherry. Immunofluorescence staining depicted that the co‐labeled ratio of c‐Fos and mCherry in the SDH of mice injected with CNO decreased significantly (Figure 5B). This finding revealed that the chemogenetic virus inhibited the CaMKIIα^+^ neuron excitation caused by Nav1.3 overexpression. Meanwhile, compared with the mice injected with saline, the paw withdrawal threshold and paw withdrawal latency of mice that had undergone the activation of hM4D with CNO was significantly increased (Figure 5C,D). These results demonstrate that increased expression of Nav1.3 in the SDH leads to the excitement of CaMKIIα^+^ neurons, which in turn induces pain. Inhibition of CaMKIIα^+^ neurons can alleviate pain induced by Nav1.3 overexpression.

### 3.5 CCI surgery downregulated spinal SIRT1 expression in mice

**FIGURE 6:**
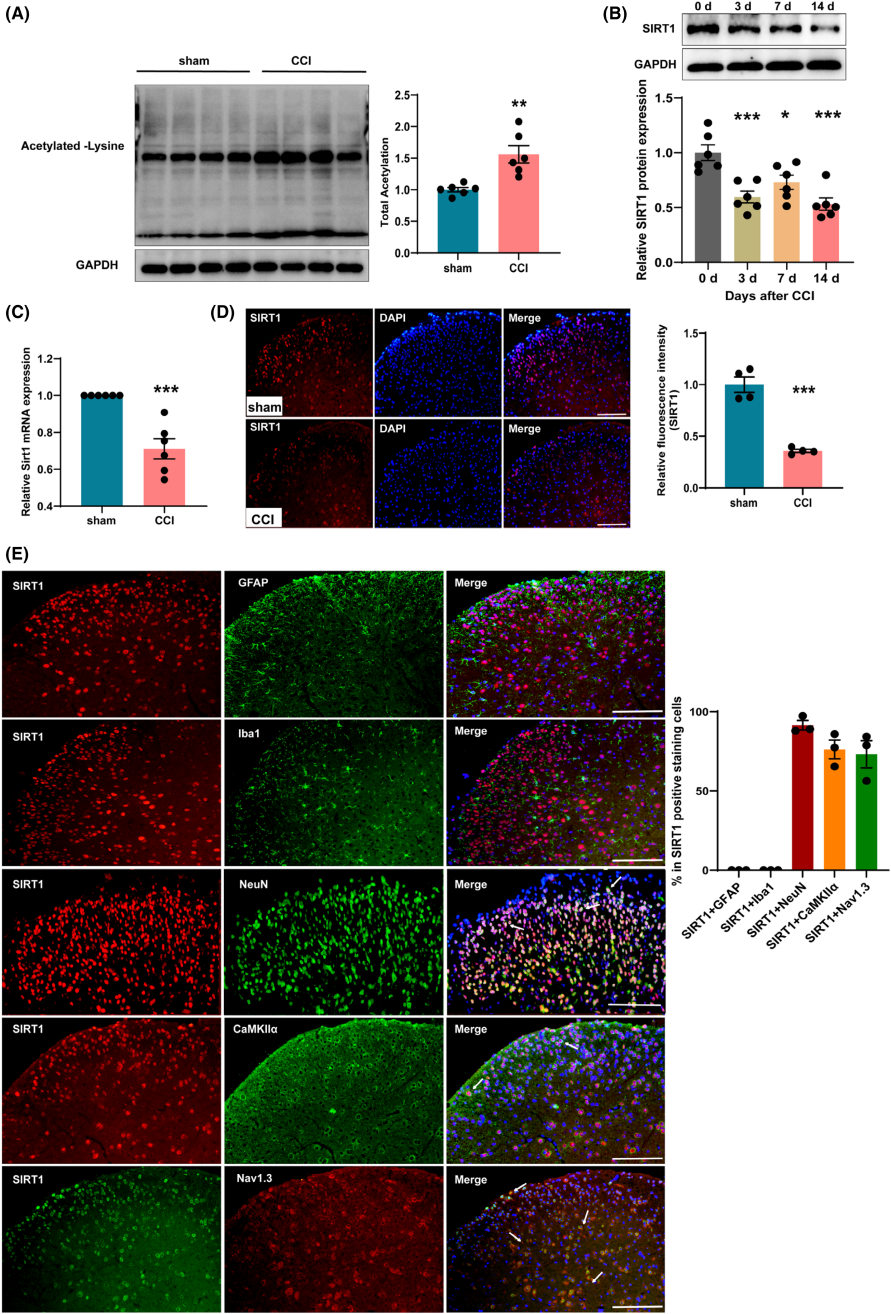
SIRT1 was decreased in the ipsilateral SDH of CCI mice. (A) Representative western blot and corresponding graphs showing that acetylated protein level increased in the SDH of CCI mice. **p < 0.01 compared with sham. (B) Representative western blot and corresponding graphs showing that SIRT1 protein level was decreased in the SDH of CCI mice. *p < 0.05 and ***p < 0.001 compared with 0 day. (C) The mRNA level of *Sirt1* was decreased in the SDH of CCI mice. ***p < 0.001 compared with sham. (D) Mean fluorescence intensity of the SIRT1 immunostaining cells decreased significantly in the SDH of CCI mice. ***p < 0.001 compared with sham. Scale bars = 200 μm. (E) Co‐detection of SIRT1 and GFAP, Iba1, NeuN, CaMKIIα, or Nav1.3 in SDH. Scale bars = 200 μm. SDH, spinal dorsal horn.

It has been reported that epigenetic modification plays a crucial role in regulating gene expression, in which the increase in histone acetylation can loosen chromatin and promote gene expression.^27^ We found that the expression of acetylated proteins was significantly increased in the SDH of CCI mice compared with the sham group (Figure 6A). On days 3, 7, and 14 after CCI, we found a significant decrease in the expression of SIRT1 protein in the ipsilateral SDH of CCI mice (Figure 6B). In addition, q‐PCR results showed that *Sirt1* mRNA level was significantly decreased at day 14 after CCI (Figure 6C). At the same time, immunofluorescence staining showed that the fluorescence signal of SIRT1 in the SDH of CCI mice was significantly reduced (Figure 6D). These results indicated a potential functional association between SIRT1 and neuropathic pain. We also identified the cell types that express SIRT1 in the spinal cord using double‐immunofluorescent staining with markers specific for neurons, astrocytes, and microglia. The results showed that SIRT1 co‐localized with NeuN, and hardly showed any overlap staining with either the GFAP or the Iba‐1. Notably, SIRT1 was found co‐localized with CaMKIIα and Nav1.3 (Figure 6E).

### 3.6 Upregulation of SIRT1 decreased Nav1.3 expression and reversed pain behavior

**FIGURE 7:**
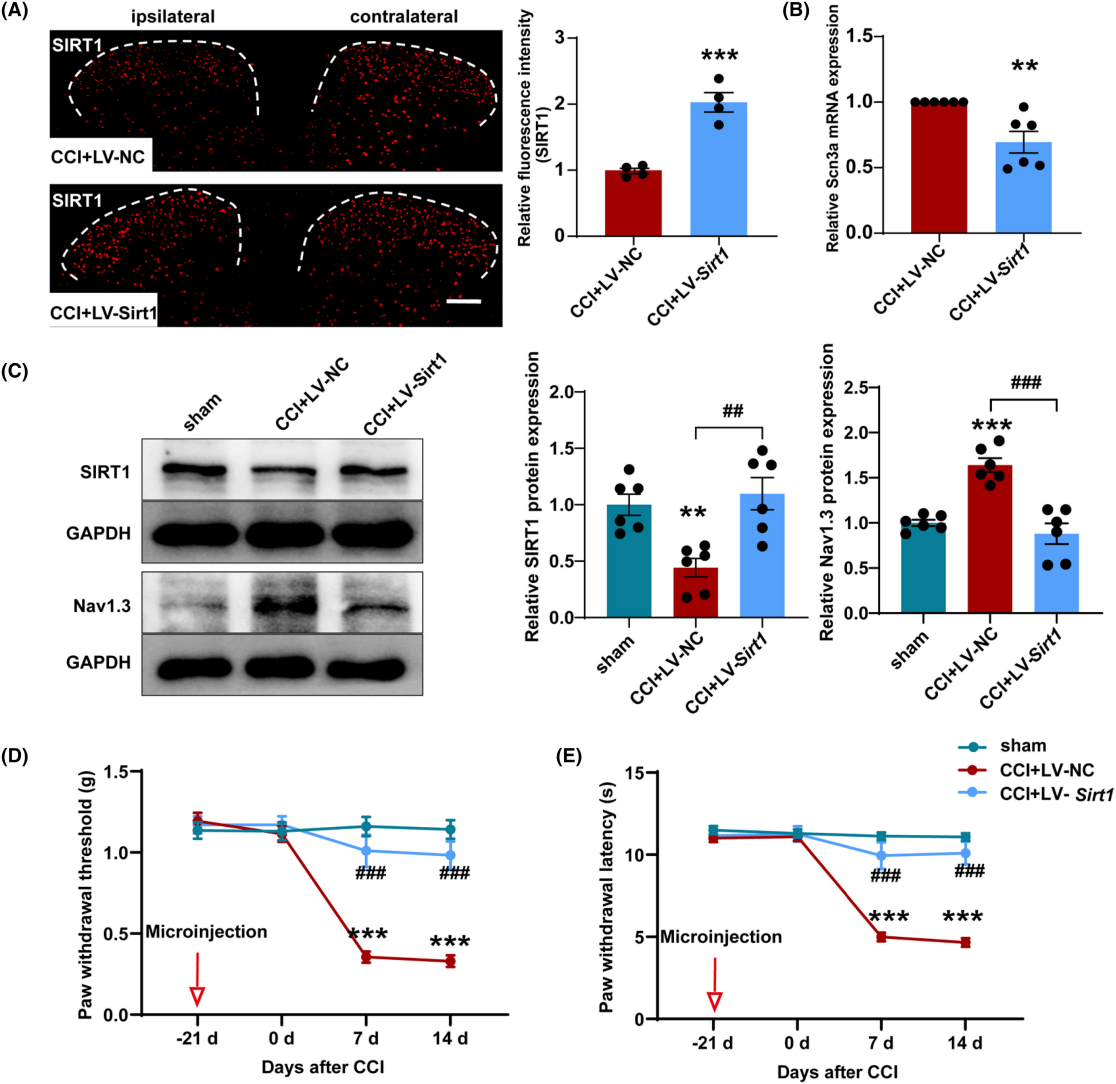
Overexpression of SIRT1 in SDH increased nociceptive thresholds in CCI mice. (A) Mean fluorescence intensity of the SIRT1 immunostaining cells increased significantly in the SDH of CCI mice after microinjection of LV‐*Sirt1*. ***p < 0.001 compared with CCI + LV‐NC. Scale bars = 200 μm. (B) mRNA level of *Scn3a* decreased in the SDH of CCI mice after microinjection of LV‐*Sirt1*. **p < 0.01 compared with CCI + LV‐NC. (C) Representative western blot and corresponding graphs show that SIRT1 protein level increased and Nav1.3 protein level decreased in the SDH of CCI mice after microinjection of LV‐*Sirt1*. **p < 0.01 and ***p < 0.001 compared with sham; ^*##*^ *p* < 0.01 and ^*###*^ *p* < 0.001 compared with CCI + LV‐NC. (D) Microinjection of LV‐*Sirt1* into the ipsilateral SDH increased paw withdrawal threshold of CCI mice. (E) Microinjection of LV‐*Sirt1* into the ipsilateral SDH increased paw withdrawal latency of CCI mice. ***p < 0.001 compared with sham; ^*###*^ *p* < 0.001 compared with CCI + LV‐NC. SDH: spinal dorsal horn.

To further investigate the role of SIRT1 in neuropathic pain and the regulation of Nav1.3, we overexpressed SIRT1 by using lentivirus LV‐*Sirt1*. We microinjected LV‐*Sirt1* and LV‐NC into the SDH of mice 21 days before CCI surgery. After 21 days of recovery, the mice then underwent CCI surgery. The position of the virus injection is on the same side as the CCI surgery. Immunofluorescence staining showed that the fluorescence signal of SIRT1 on the LV‐*Sirt1*‐injected side of SDH was significantly enhanced (Figure 7A). The q‐PCR results showed that microinjection of LV‐*Sirt1* induced remarkable down regulation of *Scn3a* mRNA in ipsilateral SDH of CCI mice (Figure 7B). Additionally, western blot results showed that the LV‐*Sirt1* group had significantly higher SIRT1 protein expression and lower Nav1.3 protein expression compared to the CCI mice injected with LV‐NC (Figure 7C). Moreover, compared with the LV‐NC group, the paw withdrawal threshold and paw withdrawal latency of CCI mice injected with lentivirus LV‐*Sirt1* were significantly increased (Figure 7D,E). These findings suggest that upregulation of SIRT1 may reverse pain behavior by decreasing the expression of Nav1.3 in CCI mice.

### 3.7 Knockdown of SIRT1 increases Nav1.3 expression and induces pain behavior

**FIGURE 8:**
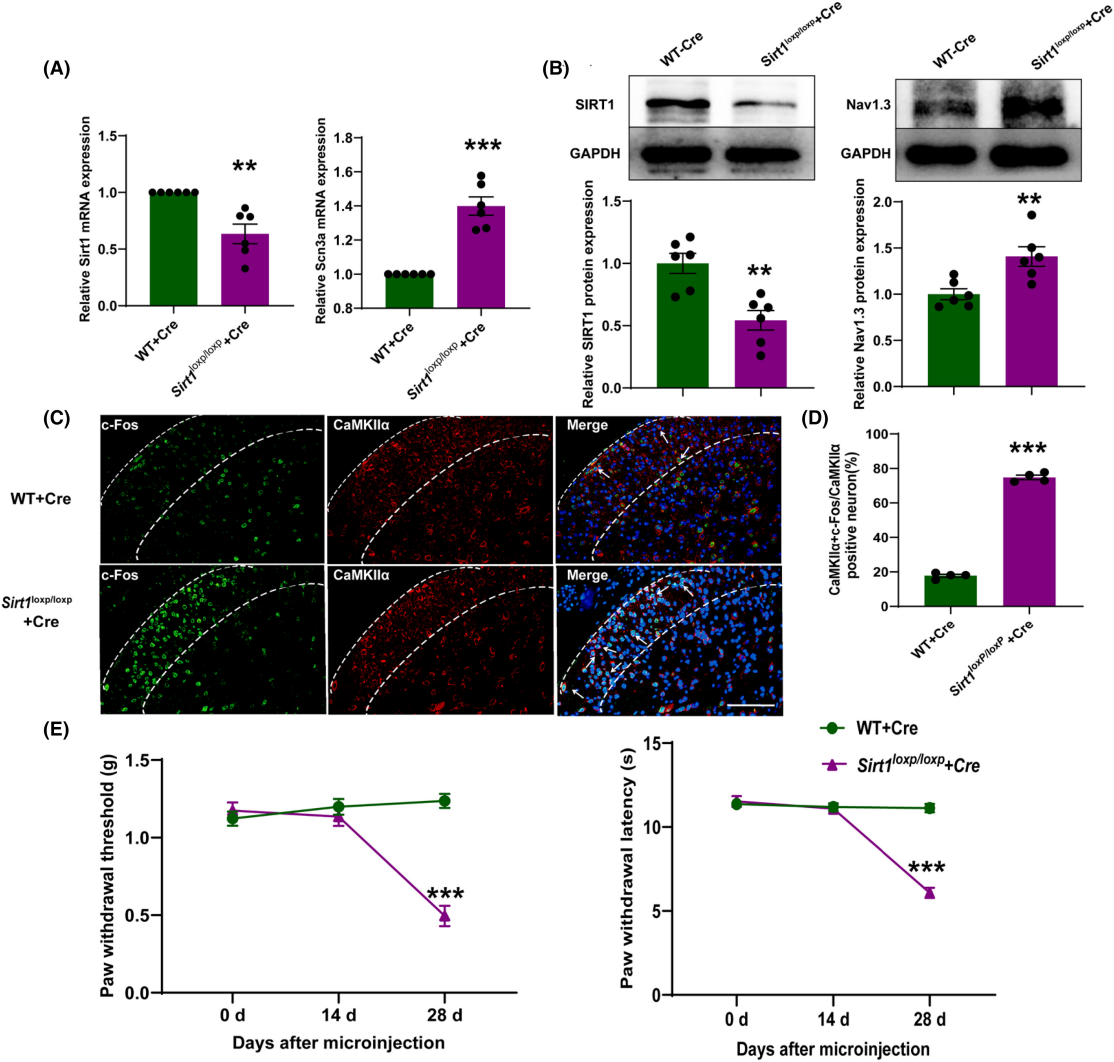
Knockdown of SIRT1 in SDH‐induced hyperalgesia. (A) mRNA level of *Sirt1* decreased and mRNA level of *Scn3a* increased in the SDH of *Sirt1* ^*loxP/loxP*^ mice after microinjection of AAV‐Cre. **p < 0.01 and ***p < 0.001 compared with WT + Cre. (B) Representative western blot and corresponding graphs show that SIRT1 protein level decreased and Nav1.3 protein level increased in the SDH of *Sirt1* ^*loxP/loxP*^ mice after microinjection of AAV‐Cre. **p < 0.01 compared with WT + Cre. (C‐D) The percentage of CaMKIIα co‐labeled with c‐Fos increased in the SDH of *Sirt1* ^*loxP/loxP*^ mice after microinjection of AAV‐Cre. ***p < 0.001 compared with WT + Cre. Scale bars = 200 μm. Blue: DAPI. (E) Microinjection of AAV‐Cre into the superficial SDH decreased paw withdrawal threshold and paw withdrawal latency of *Sirt1* ^*loxP/loxP*^ mice. ***p < 0.001 compared with WT + Cre. SDH: spinal dorsal horn.

Subsequently, SIRT1 was knocked down in *Sirt1* ^*loxP/loxP*^ mice after injecting the pAAV‐Syn‐Cre into the unilateral SDH. Microinjection of pAAV‐Syn‐Cre induced remarkable downregulation of SIRT1 mRNA and protein in *Sirt1* ^*loxP/loxP*^ mice at day 28 post‐injection, indicating successful transfection of the pAAV‐Syn‐Cre into the ipsilateral SDH. Microinjection of pAAV‐Syn‐Cre also increased the expression of *Scn3a* mRNA and Nav1.3 protein in the ipsilateral SDH compared to that of the wild‐type (WT) mice injected with pAAV‐Syn‐Cre (Figure 8A,B). These findings provide further evidence that the decreased expression of SIRT1 in the SDH may play an important role in the increased expression of Nav1.3. Furthermore, we observed that the activation of CaMKIIα^+^ neurons in the SDH was increased after knocking down of *Sirt1* (Figure 8C,D). Additionally, the paw withdrawal threshold and paw withdrawal latency were significantly decreased in *Sirt1* knockdown mice (Figure 8E). Overall, these results suggest that the pain induced by knocking down *Sirt1* in the SDH is possibly due to increased Nav1.3 expression and activation of CaMKIIα^+^ neurons.

### 3.8 Downregulation of SIRT1 induces pain by epigenetically enhancing Nav1.3 expression

**FIGURE 9:**
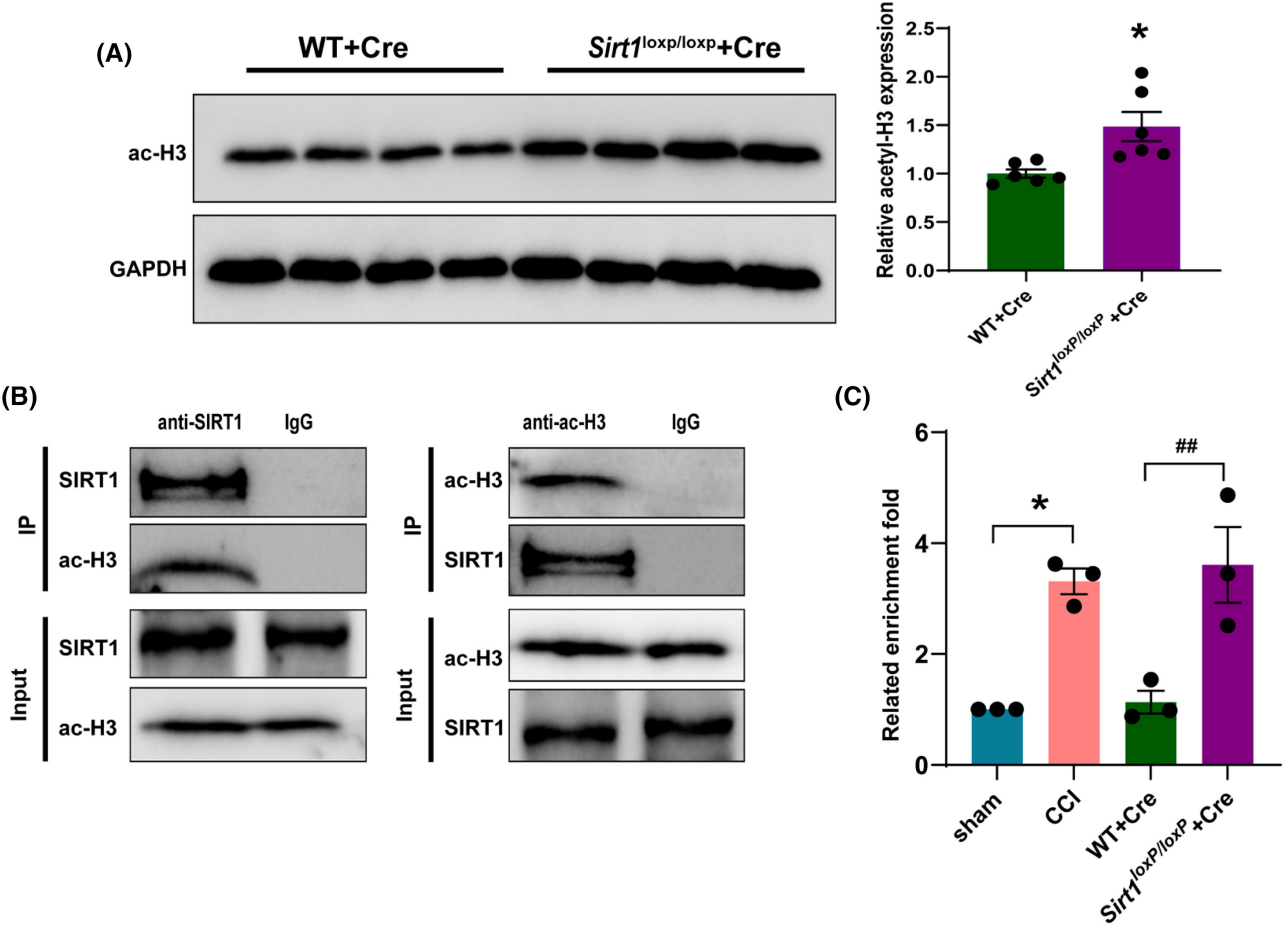
The acetylation level of histone H3 in the *Scn3a* promoter region was increased in the SDH of CCI mice and after knockdown of *Sirt1* in SDH. (A) Representative western blot and corresponding graphs showing that acetylated H3 protein level increased in the SDH of *Sirt1* ^*loxP/loxP*^ mice after microinjection of AAV‐Cre. *p < 0.05 compared with WT + Cre. (B) SIRT1 was immunoprecipitated with acetylated H3 in SDH of mice. (C) The level of acetylated H3 in the *Scn3a* promoter region (−529 to −139 bp) upstream of the transcription start site was increased in the SDH of CCI mice and after knockdown of SIRT1 in SDH (*n* = 3, 12 mice in total); *p < 0.05 compared with sham; ^*##*^ *p* < 0.01 compared with WT + Cre.

Next, we investigated the mechanism by which SIRT1 regulates Nav1.3 expression. We knocked down SIRT1 in the SDH and observed that the level of acetylated histone H3 was increased significantly in *Sirt1* ^*loxP/loxP*^ mice as compared to WT mice (Figure 9A). Co‐immunoprecipitation result has shown that the SIRT1 protein has a binding relationship with the ac‐H3 protein in the SDH of mice (Figure 9B). Furthermore, we also performed ChIP assays to determine H3 acetylation levels at *Scn3a* promoter regions. We found that H3 acetylation levels in the region of −529 to −139 bp upstream of the transcription start site of *Scn3a* were significantly increased in CCI mice and *Sirt1* ^*loxP/loxP*^ mice with knockdown of *Sirt1* (Figure 9C). These results indicate that downregulation of SIRT1 induces pain by increasing histone acetylation at the *Scn3a* promoter regions and subsequently increasing transcription.

## 4 DISCUSSION

Our study reveals that a decline in SIRT1 within spinal CaMKIIα^+^ neurons is associated with the development of pain subsequent to nerve injury, involving the Nav1.3 sodium channel. The results suggest that CaMKIIα^+^ neurons become overactive after CCI and that inhibiting their activation can relieve CCI‐induced pain. Furthermore, we observed a decrease in SIRT1 and an increase in Nav1.3 in ipsilateral SDH of mice after CCI. Over expression of SIRT1 alleviated pain and reversed the increase in Nav1.3, while knockdown of SIRT1 or overexpression of Nav1.3 promoted CaMKIIα^+^ neuron activation and induced pain. We also found that knockdown of SIRT1 increased the acetylation level of histone H3 in the *Scn3a* promoter region, which promoted the expression of Nav1.3.

Neuropathic pain stemming from somatic nervous system injuries or lesions often persists for extended periods. Patients typically experience heightened sensitivity to nonpainful stimuli (allodynia), increased sensitivity to painful stimuli (hyperalgesia), and spontaneous pain.^28^ Central sensitization is a key characteristic of chronic pain,^29^, ^30^ with the spinal cord serving as the primary pathway for transmitting peripheral pain signals to the brain.^31^, ^32^ The spinal cord's response to pain signals depends on various mechanisms that can amplify or dampen the activity of dorsal horn neurons. Following nerve damage, alterations in these mechanisms lead to heightened neuronal responses to incoming signals, ultimately resulting in increased pain through enhanced output to higher centers.^33^ The Ca^2+^/calmodulin‐dependent protein kinase II is a highly conserved serine/threonine kinase. Research indicates that the alpha subunit of CaMKII is specifically localized to excitatory synapses of forebrain neurons, while it is not expressed in inhibitory GAD/GABA‐positive cells throughout the forebrain, brainstem, and spinal cord.^34^ Studies have shown that upregulation of the nonselective cation channel TRPV1 in SDH sensory neurons after nerve injury activates CaMKII, leading to increased neuronal excitability and pain induction.^35^ Moreover, the use of the CaMKII inhibitor KN93 has been found to alleviate pain behavior.^36^ These studies suggest that the excitation of CaMKIIα^+^ neurons in the SDH may play an important role in pain. However, there have been very limited studies on the distribution and localization of CaMKIIα in the SDH. It has been observed that nearly all inhibitory and excitatory neurons in the superficial layer of the SDH exhibit both CaMKIIα and CaMKIIβ immunoreactivity.^37^ Here, we found that CaMKIIα co‐stained with the excitatory neuron marker, but hardly with the GABAergic neuron marker. This result is consistent with Benson's report that GABA immunoreactive neurons in the dorsal horn lacked CaMKIIα immunoreactivity.^38^ We think that the differences in CaMKIIα expression may be related to the selection of antibodies and the fixation of tissues. The precise cellular localization of CaMKIIα in the spinal cord requires further verification. We also found that the activation of CaMKIIα^+^ neurons in the SDH increased after CCI. Pain was relieved after inhibition of CaMKIIα^+^ neurons by chemogenetic virus. This suggests that activation of CaMKIIα^+^ neurons is the key factor in pain development. In this regard, we speculate that pain after nerve injury is mainly triggered by the activation of excitatory neurons expressing CaMKIIα.

Neuronal excitability, synaptic transmission, and action potential propagation are closely linked to ion channels.^39^ Abnormal increases in sodium channel activity are implicated in central neuronal hyperexcitability associated with conditions such as epilepsy, chronic pain, neurodegenerative diseases, and spasticity.^40^ To explore the mechanism by which CaMKIIα neurons are activated during pain, we performed transcriptome sequencing of mice SDH tissues. We found that among the differentially expressed ion channel genes in CCI mice, the *Scn3a* gene encoding the Nav1.3 sodium channel was significantly increased. Nav1.3, a voltage‐gated sodium channel in the cell membrane, is involved in action potential generation and conduction in excitable cells and is implicated in various neurological disorders.^41^ Previous studies have demonstrated that functional sodium channel currents in lamina I/II neurons primarily consist of the Nav1.2 and Nav1.3 isoforms.^42^ Additionally, abnormal expression of Nav1.3 in second‐order spinal cord dorsal horn neurons and third‐order thalamic neurons along the pain pathway to SCI has been reported, leading to hyperexcitability and amplification of pain signals.^43^ Furthermore, intrathecal administration of antisense oligodeoxynucleotides (ODNs) targeting Nav1.3 has been shown to reduce hyperexcitability of dorsal horn neurons, as well as alleviate mechanical allodynia and thermal hyperalgesia after spinal cord injury.^44^ Therefore, the increased expression of Nav1.3 may be responsible for the activation of CaMKIIα^+^ neurons during pain. Our study found that the expressions of Nav1.3 protein and S*cn3a* mRNA increased in the SDH after CCI, and Nav1.3 co‐labeled with CaMKIIα. Knockdown of Nav1.3 in the SDH using LV‐*Scn3a* shRNA alleviated CCI‐induced pain. Conversely, overexpression of Nav1.3 by LV‐*Scn3a* increased CaMKIIα^+^ neuron activation and caused hyperalgesia in naive mice. To further validate the mechanism of increased Nav1.3‐induced pain, we also used chemogenetic virus to inhibit CaMKIIα^+^ neuron activation while overexpressing Nav1.3 in the SDH. The results showed that inhibition of CaMKIIα^+^ neurons prevented the pain induced by overexpression of Nav1.3. These findings suggest that increased expression of Nav1.3 in the SDH causes pain by activating CaMKIIα^+^ neurons.

Epigenetic mechanisms may be pivotal in regulating the development of chronic pain. SIRT1, a nicotine adenine dinucleotide (NAD+)‐dependent histone deacetylase, governs diverse cellular functions such as DNA repair, inflammatory response, cell cycle, and apoptosis.^45^ Activation of SIRT1 has emerged as a potential approach to prevent or treat neuropathic pain.^46^ Evidence indicates that SIRT1‐mediated epigenetic control of mGluR1/5 expressions plays a significant role in rat DNP.^47^ Likewise, our study also confirmed the involvement of SIRT1 in CCI‐induced pain and discovered a new mechanism explaining how the absence of SIRT1 causes pain. Moreover, our findings showed that SIRT1 protein and *Sirt1* mRNA expression were significantly decreased in the SDH of CCI mice and that SIRT1 co‐labeled with both CaMKIIα and Nav1.3. Based on this, we hypothesized that SIRT1 might regulate Nav1.3 expression. When we overexpressed SIRT1 in the SDH using LV‐*Sirt1*, the CCI‐induced increase in Nav1.3 expression was reversed, and CCI‐induced pain was alleviated. Conversely, knocking down *Sirt1* in the SDH increased Nav1.3 expression and CaMKIIα^+^ neurons activation, and reduced pain threshold in mice. The co‐immunoprecipitation result showed that SIRT1 protein has a binding relationship with ac‐H3 protein in the SDH of mice. In addition, The CHIP assay revealed that histone H3 acetylation in the *Scn3a* promoter region was significantly increased after CCI and after SIRT1 knockdown. These findings suggest that SIRT1 epigenetically regulates Nav1.3 expression, influencing the activation of CaMKIIα^+^ neurons and contributing to the pain process.

While the present study sheds light on the subject, it is important to acknowledge its limitations. First, there is a lack of electrophysiological data that directly exhibit the excitability of spinal CaMKIIα^+^ neurons, and whole‐cell patch‐clamp experiments are needed for recording neurons in spinal cord slices. Second, it is necessary to clarify the upstream regulators of SIRT1 expression in CCI mice. According to previous studies, the expression of SIRT1 is subject to many layers of regulation, including at the transcriptional and post‐transcriptional. For instance, transcription factors and co‐factors such as P53, C‐MYC, and FOXO3a were reported to repress or promote SIRT1 transcription. SIRT1 is also post‐transcriptionally regulated by noncoding RNAs, especially microRNAs (miRNAs). Several miRNAs (miR‐34, miR‐133, miR‐199, miR‐212, and miR‐448) downregulate SIRT1 protein expression by binding to the 3′‐untranslated regions of SIRT1.^48^ However, additional experiments are needed to explore whether the specific mechanism of the downregulation of spinal SIRT1 involves transcription factors and miRNAs as mentioned above.

In conclusion, the results of the study suggest that the downregulation of SIRT1 in the SDH, following CCI, leads to increased histone H3 acetylation in the *Scn3a* promoter region. This promotes the expression of Nav1.3, which subsequently causes CaMKIIα^+^ neuron activation, resulting in pain. Alleviating pain by reversing SIRT1 downregulation can be a potential therapeutic target for local spinal cord analgesics.

## AUTHOR CONTRIBUTIONS

Yuanzeng Wang: methodology, formal analysis, visualization, and writing—original draft. Yidan Zhang: investigation and writing—review & editing. Nan Ma: investigation. Wen Zhao: investigation. Xiuhua Ren: project administration and Validation. Yanyan Sun: resources and writing—review & editing. Weidong Zang: resources, supervision, and validation. Jing Cao: conceptualization, funding acquisition, resources, supervision, and writing – review & editing.

## FUNDING INFORMATION

This work was supported by grants from the National Natural Science Foundation of China (Grant number 82371237) to J. Cao and the Program for Innovative Research Team in Universities of Henan Province (22IRTSTHN028).

## CONFLICT OF INTEREST STATEMENT

The authors declare no conflict of interest.

## Supporting information


Data S1

 

## Data Availability

The data that support the findings of this study are available from the corresponding author upon request.
