# Supplementary material for: SIRT1 mediates the excitability of spinal CaMKIIα‐positive neurons and participates in neuropathic pain by controlling Nav1.3
Source: CNS Neurosci Ther. 2024 Jun 3;30(6):e14764. doi: 10.1111/cns.14764 (PMC11145124; doi:10.1111/cns.14764)
Supplement: Supplementary file 2 [file CNS-30-e14764-s001.docx]

Supplementary figure 1. Full unedited gel/blot for Figure 3E. Representative western blot showing that Nav1.3 protein level was increased in the SDH of CCI mice.

Supplementary figure 2. Full unedited gel/blot for Figure 4A. Representative western blot showing that Nav1.3 protein level decreased in the SDH of CCI mice after microinjection of LV-*Scn3a* shRNA.

Supplementary figure 3. Full unedited gel/blot for Figure 4D. Representative western blot showing that Nav1.3 protein level increased in the ipsilateral SDH of naïve mice after microinjection of LV-*Scn3a*.

Supplementary figure 4. Full unedited gel/blot for Figure 6A. Representative western blot showing that acetylated protein level increased in the SDH of CCI mice.

Supplementary figure 5. Full unedited gel/blot for Figure 6B. Representative western blot showing that SIRT1 protein level was decreased in the SDH of CCI mice.

Supplementary figure 6. Full unedited gel/blot for Figure 7C. Representative western blot showing that SIRT1 protein level increased and Nav1.3 protein level decreased in the SDH of CCI mice after microinjection of LV-*Sirt1*.

Supplementary figure 7. Full unedited gel/blot for Figure 8B. Representative western blot showing that SIRT1 protein level decreased and Nav1.3 protein level increased in the SDH of *Sirt1*^loxP/loxP^ mice after microinjection of AAV-Cre.

Supplementary figure 8. Full unedited gel/blot for Figure 9A. Representative Western blot showing that acetylated H3 protein level increased in the SDH of *Sirt1*^loxP/loxP^  mice after microinjection of AAV-Cre.

Supplementary figure 9. Full unedited gel/blot for Figure 9B. Representative Western blot showing that SIRT1 was immunoprecipitated with acetylated H3 in SDH of mice.
