# Supplementary material for: SIRT1 mediates the excitability of spinal CaMKIIα‐positive neurons and participates in neuropathic pain by controlling Nav1.3
Source: CNS Neurosci Ther. 2024 Jun 3;30(6):e14764. doi: 10.1111/cns.14764 (PMC11145124; doi:10.1111/cns.14764)

Full unedited gel/blot for Figure 3E

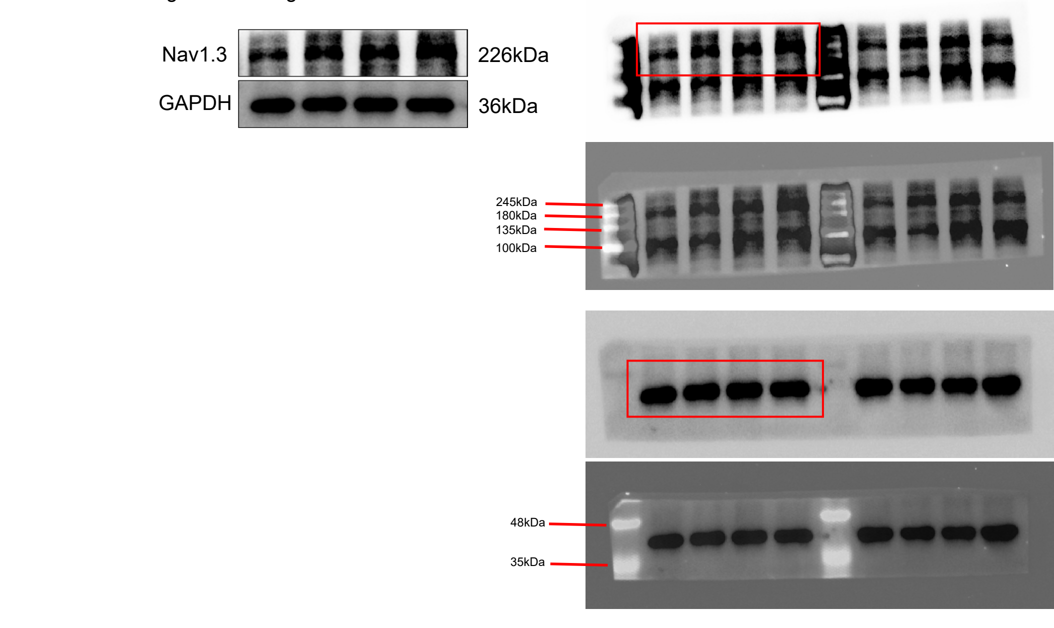

Full unedited gel/blot for Figure 4A

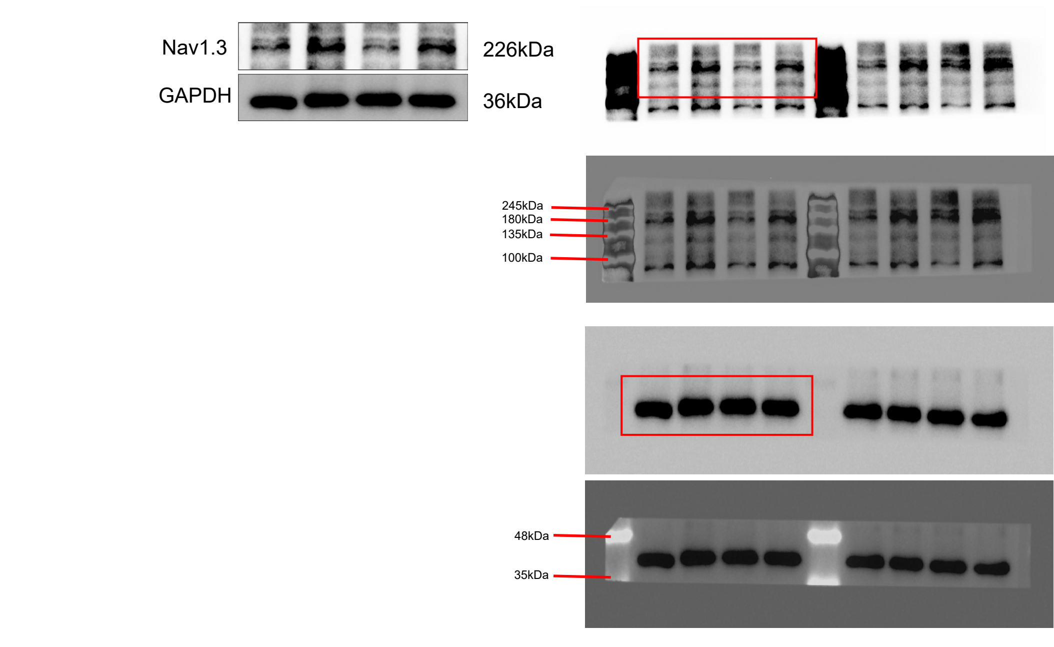

Full unedited gel/blot for Figure 4C

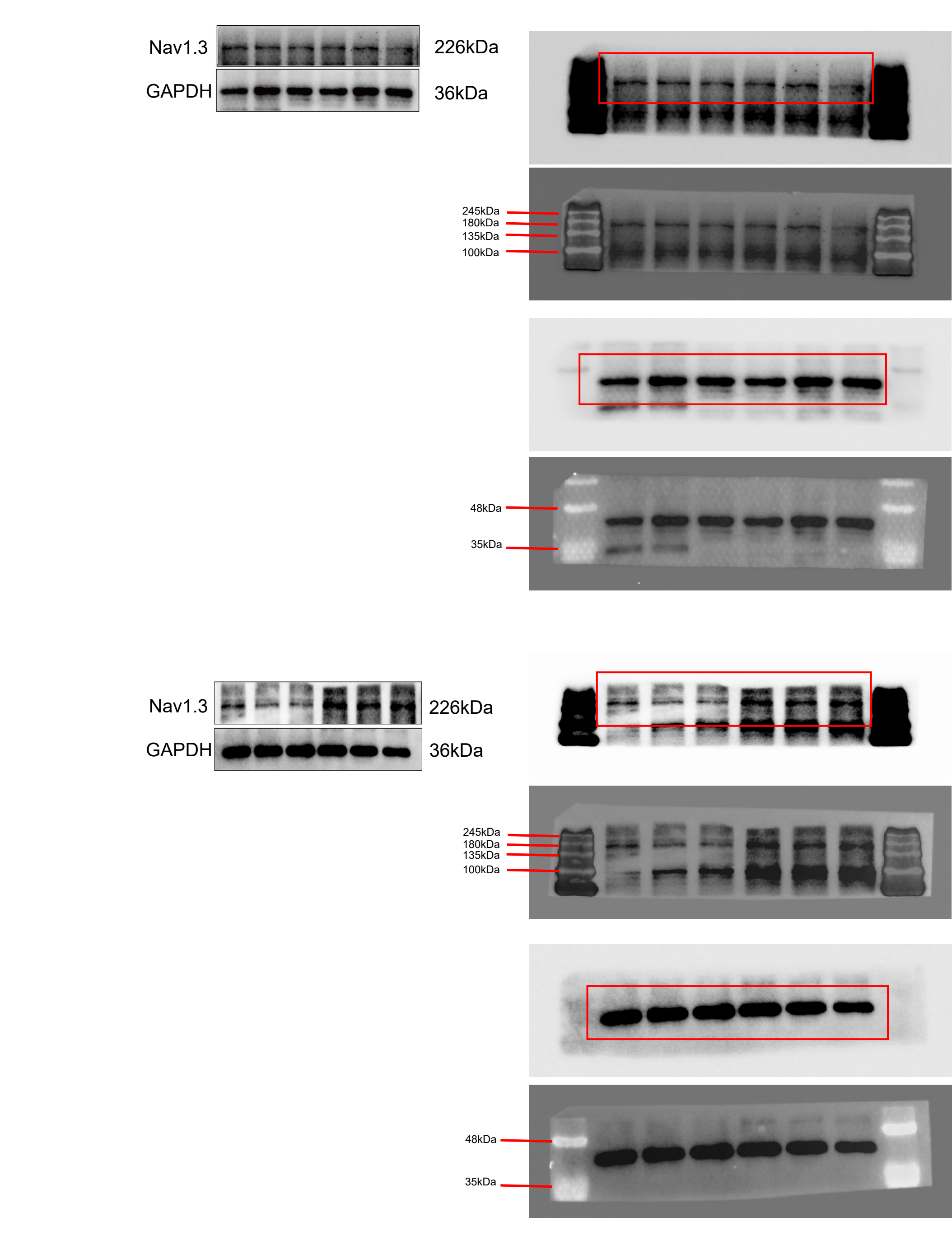

Full unedited gel/blot for Figure 6A

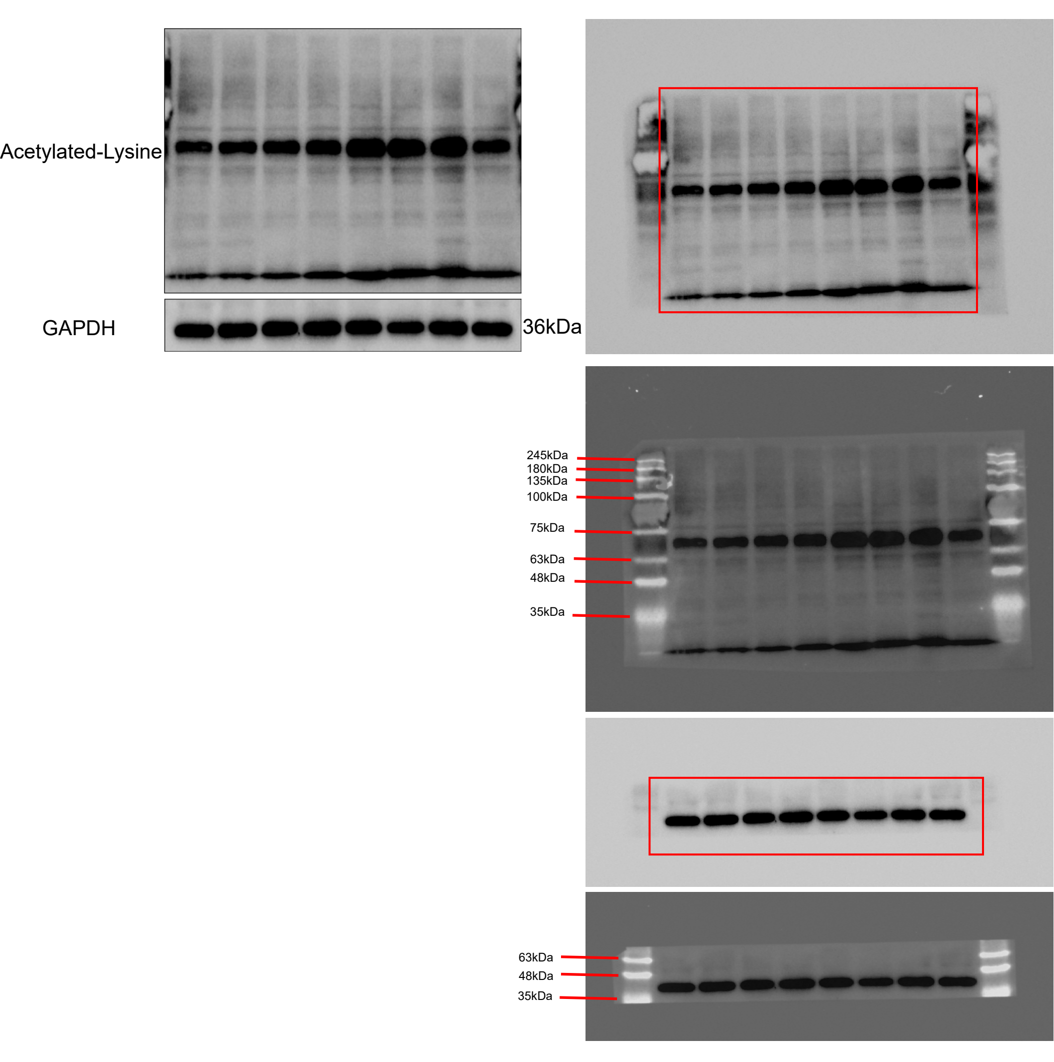

Full unedited gel/blot for Figure 6B

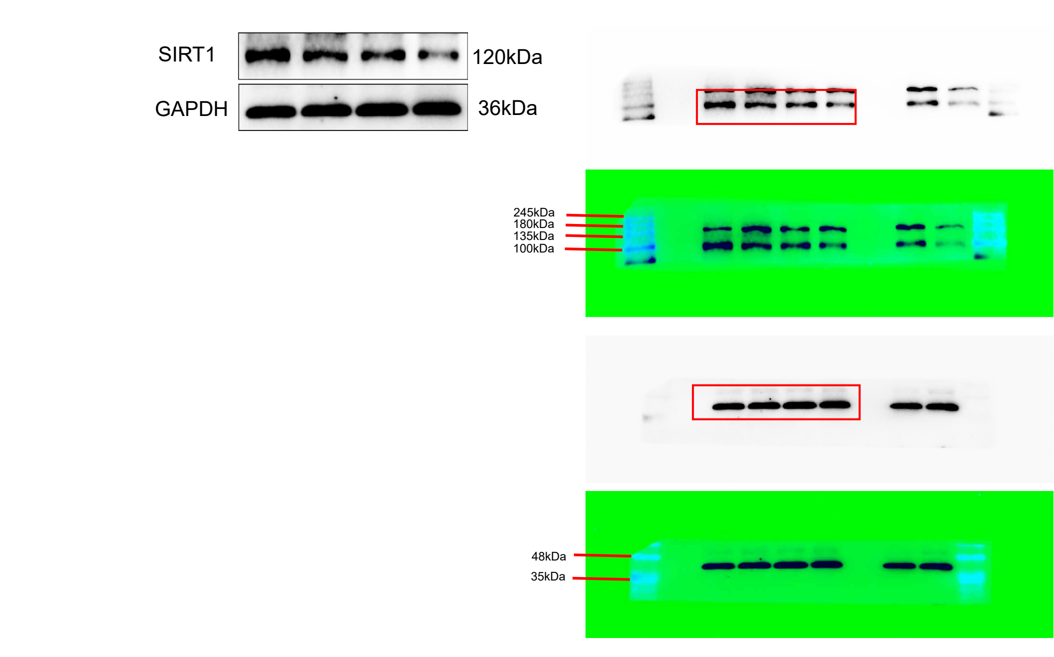

Full unedited gel/blot for Figure 7C

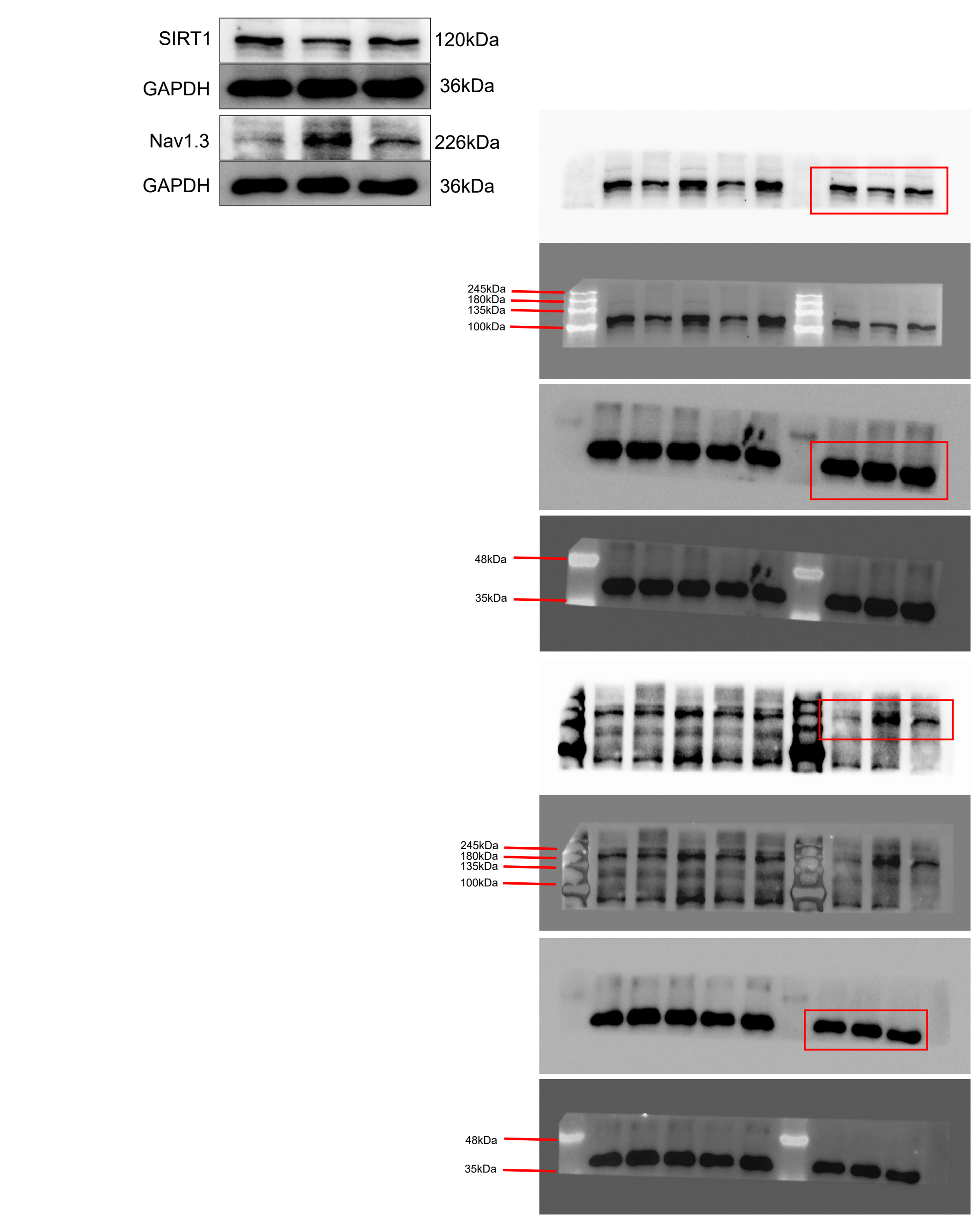

Full unedited gel/blot for Figure 8B

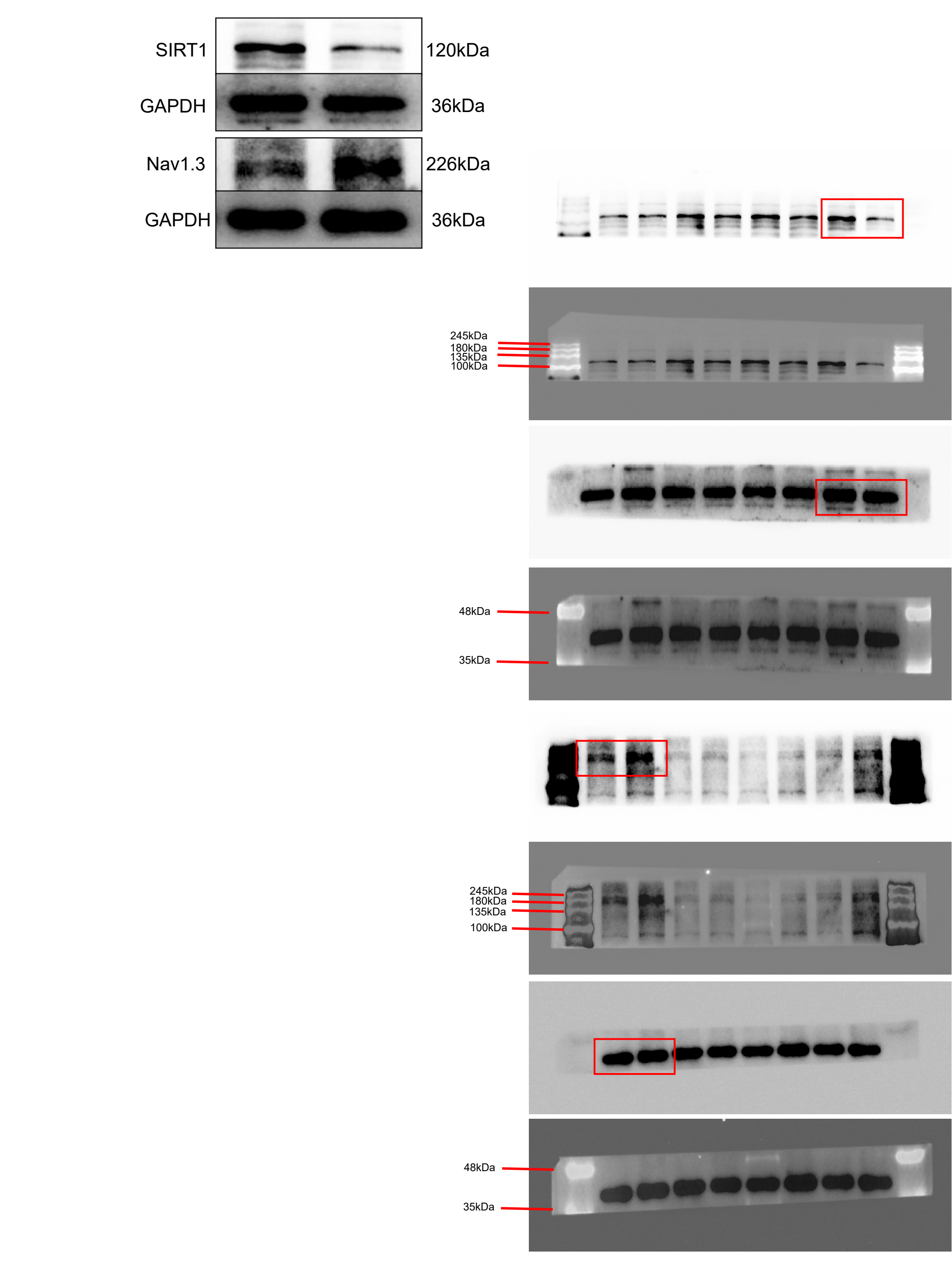

Full unedited gel/blot for Figure 9A

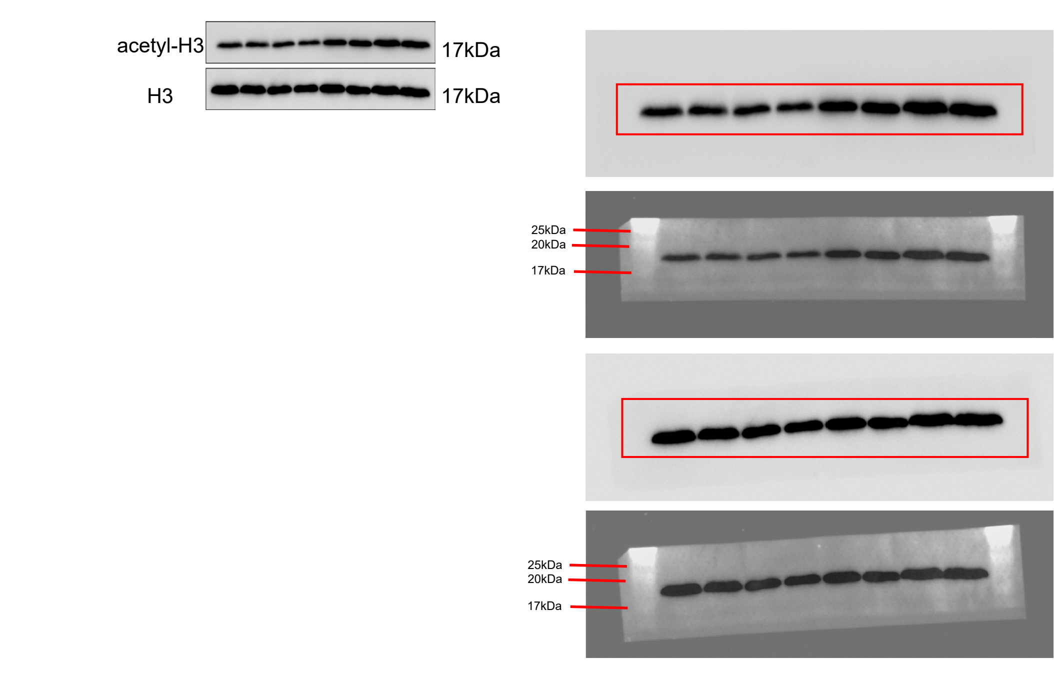

Full unedited gel/blot for Figure 9B

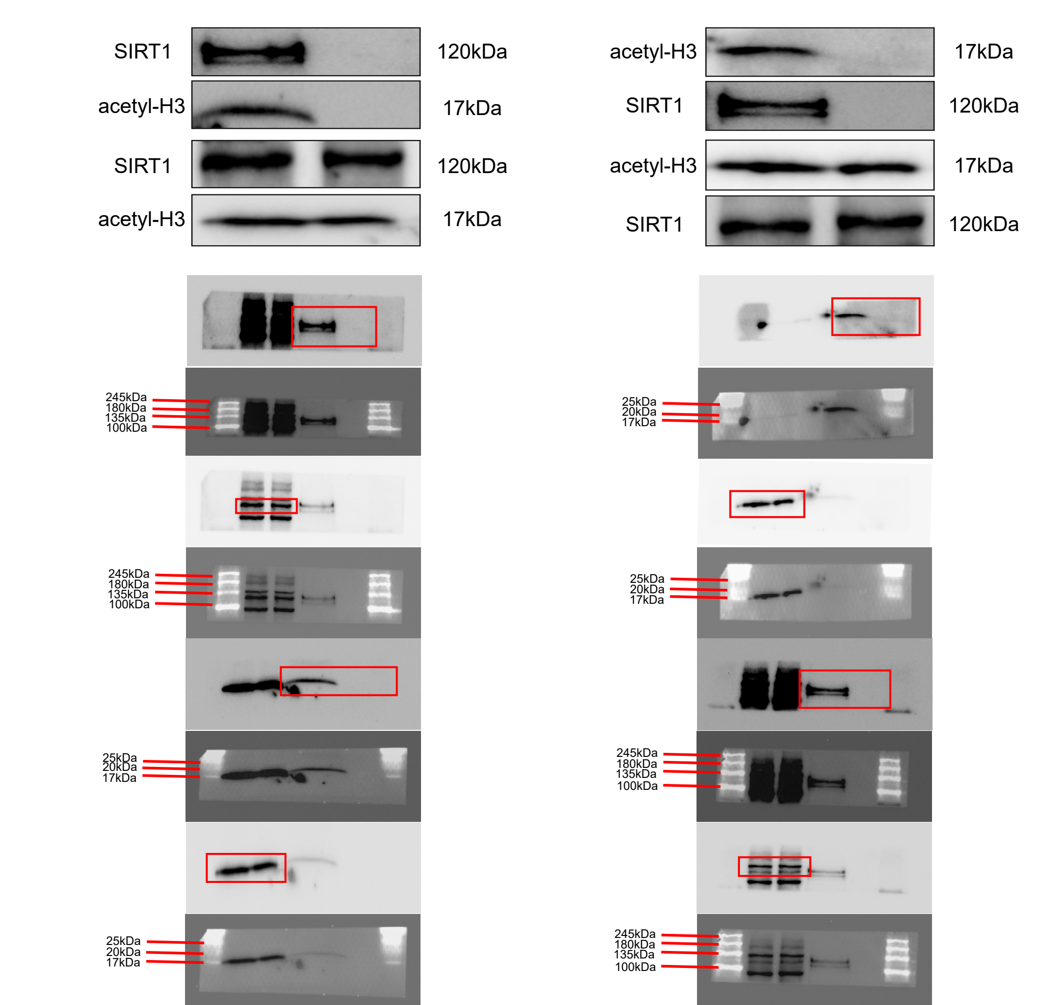

Supplement: Supplementary file 1 — Data S1 [file CNS-30-e14764-s002.pdf]
